# Critical transition of urban ozone formation regime in the North China Plain

**DOI:** 10.1093/nsr/nwaf596

**Published:** 2026-01-02

**Authors:** Likun Xue, Yujiao Zhu, Jian Gao, Xuelian Zhong, Can Cui, Shuai Wang, Zhiwen Jiang, Yue Sun, Qinyi Li, Yuqiang Zhang, Hong Li, Yingnan Zhang, Shanshan Wang, Min Zhao, Hengqing Shen, Yujie Zhang, Guigang Tang, Tao Wang, Wenxing Wang

**Affiliations:** Environment Research Institute, Shandong University, Qingdao 266237, China; Environment Research Institute, Shandong University, Qingdao 266237, China; State Key Laboratory of Environmental Criteria and Risk Assessment, Chinese Research Academy of Environmental Sciences, Beijing 100012, China; Environment Research Institute, Shandong University, Qingdao 266237, China; Environment Research Institute, Shandong University, Qingdao 266237, China; Department of Ambient Air Quality Monitoring, China National Environmental Monitoring Centre, Beijing 100012, China; Shanghai Key Laboratory of Atmospheric Particle Pollution and Prevention (LAP^3^), Department of Environmental Science and Engineering, Fudan University, Shanghai 200438, China; Environment Research Institute, Shandong University, Qingdao 266237, China; Environment Research Institute, Shandong University, Qingdao 266237, China; Environment Research Institute, Shandong University, Qingdao 266237, China; State Key Laboratory of Environmental Criteria and Risk Assessment, Chinese Research Academy of Environmental Sciences, Beijing 100012, China; Environment Research Institute, Shandong University, Qingdao 266237, China; Shanghai Key Laboratory of Atmospheric Particle Pollution and Prevention (LAP^3^), Department of Environmental Science and Engineering, Fudan University, Shanghai 200438, China; Environment Research Institute, Shandong University, Qingdao 266237, China; Environment Research Institute, Shandong University, Qingdao 266237, China; State Key Laboratory of Environmental Criteria and Risk Assessment, Chinese Research Academy of Environmental Sciences, Beijing 100012, China; Department of Ambient Air Quality Monitoring, China National Environmental Monitoring Centre, Beijing 100012, China; Department of Civil and Environmental Engineering, The Hong Kong Polytechnic University, Hong Kong 999077, China; Environment Research Institute, Shandong University, Qingdao 266237, China; State Key Laboratory of Environmental Criteria and Risk Assessment, Chinese Research Academy of Environmental Sciences, Beijing 100012, China

**Keywords:** ozone pollution, urban air quality, atmospheric photochemistry, North China Plain

## Abstract

Over the past decade, China has achieved remarkable progress in mitigating aerosol pollution. However, ozone (O_3_) pollution still shows a worsening trend, implying that China’s control measures may have been less effective in tackling O_3_ pollution. Herein, we conduct synchronized observations of O_3_ and its precursors across 37 cities in the heavily polluted North China Plain (NCP) during the summer of 2021 and apply a unified observation-based model to diagnose O_3_ formation mechanisms. Our results reveal a significant transition in the urban O_3_ formation regime, shifting from being primarily volatile organic compound (VOC) limited to VOC–nitrogen oxides (NO_x_) co-limited across the NCP between the 2010s and the 2020s. Notably, the primary VOC species, their respective sources, and the optimal VOCs/NO_x_ reduction ratios exhibit remarkable regional consistency. Modeling analyses further indicate that a long-term national ‘carbon neutrality’ strategy could effectively alleviate O_3_ pollution, with targeted VOC emission reductions from major anthropogenic sources offering the greatest mitigation potential. These findings underscore the efficacy of China’s endeavors in mitigating O_3_ pollution, although the effects are not immediately evident from ambient O_3_ concentrations. O_3_ pollution control in Chinese cities has reached a critical inflection point, offering considerable flexibility and feasibility in formulating future control policies.

## Teaser

Urban ozone formation in North China has reached a critical turning point, shifting from a predominantly VOC-limited regime to a VOCNOx co-limited regime across cities. This transition indicates that targeted reductions in key anthropogenic VOC emissions, aligned with the ``Carbon Neutrality’ strategy, can enable more effective and flexible O_3_ pollution control.

## INTRODUCTION

Ozone (O_3_) pollution poses a significant challenge to air quality management in China. Over the last decade, the implementation of the ‘Air Pollution Prevention and Control Action Plan (2013–2017)’ and the subsequent ‘Three-year Action Plan to Fight Air Pollution (2018–2020)’ has resulted in substantial declines in the ambient concentrations of particulate matters (PM_2.5_ and PM_10_), nitrogen oxides (NO_x_), sulfur dioxide (SO_2_) and carbon monoxide (CO) [1]. Anthropogenic NO_x_ emissions have been significantly reduced, by over 30% from 2012 to 2020, with a delayed and less pronounced reduction in the emissions of volatile organic compounds (VOCs) from 2016 to 2020 (9%) [2]. Despite these notable efforts, the national average levels of O_3_ continued to rise from 2013 to 2019 and have subsequently fluctuated at elevated values, even amid the implementation of stricter O_3_ pollution control measures and the influence of the COVID-19 pandemic [3]. Thus far, no city in China has attained the World Health Organization (WHO) recommended standard for the maximum daily 8-h average O_3_ concentration (MDA8 O_3_; 100 μg/m^3^), with the O_3_ levels in many cities exceeding the limit by over 50% [4]. Therefore, mitigating O_3_ pollution is an important aspect of improving air quality in China, particularly within the framework of the ‘Beautiful China Initiative’ [5].

The challenge of O_3_ pollution control arises from the highly non-linear relationship between O_3_ and its precursors (mainly NO_x_ and VOCs), alongside the necessity of regional coordination for precursor emission control [6,7]. Typically, given the varying regional distributions and chemical lifetimes of NO_x_ and VOCs, O_3_ production in urban areas is primarily constrained by VOCs and saturated with NO_x_ [8,9]. Under such conditions, reducing NO_x_ emissions by a certain amount exacerbates O_3_ concentrations. Previous studies have attributed the deteriorating O_3_ pollution across China to the reduction of anthropogenic NO_x_ emissions [10–13], although the concurrent PM_2.5_ reduction might also contribute by weakening the heterogeneous loss of HO_2_ radicals on the aerosol surfaces [11]. Furthermore, owing to the non-linear O_3_–precursor relationship, determining the optimal VOCs/NO_x_ emission reduction ratio is crucial for formulating effective O_3_ pollution mitigation policies. However, the optimal VOCs/NO_x_ reduction ratios vary significantly among different cities under the VOC-limited (and NO_x_-saturated) regime [14,15]. Coupled with complex regional transport mechanisms, this poses considerable challenges to effective regional coordination aimed at mitigating O_3_ pollution on a larger spatial scale. The ultimate strategy to control O_3_ pollution involves appropriately reducing the emissions of NO_x_ and VOCs to shift O_3_ formation into an NO_x_-limited regime [16]. In areas where O_3_ formation is limited by VOCs and saturated with NO_x_, the O_3_ pollution control process usually includes a critical inflection point, across which a transition occurs from a VOC-limited to a VOC–NO_x_ co-limited regime, alongside increasing O_3_ concentrations. Identifying this transition is essential for developing long-term strategies to mitigate O_3_ pollution.

Here, we conducted a large-scale field campaign simultaneously across 37 cities in the North China Plain (NCP), the region with the most severe O_3_ pollution in China, from June to August 2021. To the best of our knowledge, this marks the first synchronized, intensive field measurement endeavor on such an extensive temporal and spatial scale, targeting photochemical air pollution in China. Our results reveal that O_3_ pollution control in the NCP region has reached a critical inflection point, as most urban areas have shifted from a VOC-limited regime in the 2010s to a VOC–NO_x_ co-limited regime in the 2020s. The dominant reactive VOC species, their emission sources, and the optimal VOCs/NO_x_ emission reduction ratios show remarkable homogeneity among individual cities, indicating that achieving coordinated regional control of O_3_ pollution has become feasible and effective. We also evaluated the future prospects for air quality improvement under China’s ‘carbon neutrality’ policy, demonstrating that national emission reduction strategies can substantially mitigate O_3_ pollution. This study demonstrates the effectiveness of China’s efforts in controlling O_3_ pollution, albeit with effects that are not immediately apparent from the ambient concentrations. Future O_3_ control policy will offer more flexibility, feasibility and optimistic prospects.

## RESULTS AND DISCUSSION

### Intense O_3_ pollution and transition of O_3_ formation regime across the NCP

**Figure 1.**
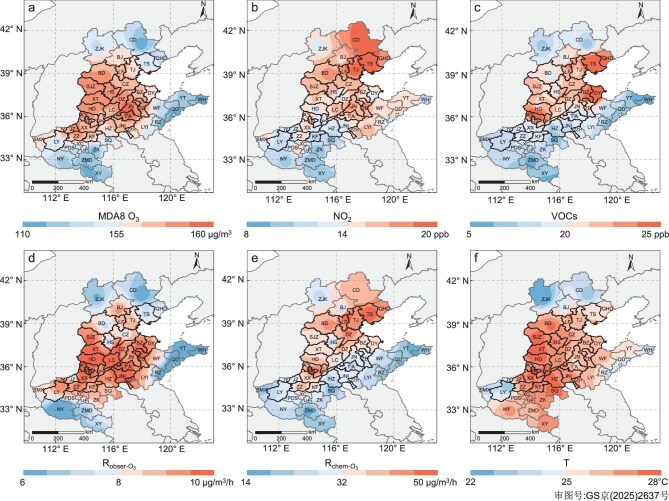
Spatial distributions of O_3_, O_3_ precursors, O_3_ concentration change rate, *in situ* O_3_ formation chemistry, and temperature in the NCP region from 1 June to 31 August 2021. (a) MDA8 O_3_ concentrations, (b) NO_2_ concentrations, (c) VOC concentrations, (d) observed rate of change in O_3_ concentrations (R_obser-O3_), (e) *in situ* net O_3_ production rate (R_chem-O3_) calculated using the observation-based model (OBM) and (f) temperature (T). The data in (d and e) were calculated between 08:00 and 17:00 (local time). All plots show the median of the daily average and are created based on spatial interpolation of the data in each city. Areas with solid black borders denote the 26 cities that failed to meet the national Grade II standards (160 μg/m^3^ for MDA8 O_3_). Refer to Table S1 for the abbreviations of the cities.

In June–August 2021, intensive field observations were concurrently conducted across 37 cities within the NCP region, encompassing Beijing and Tianjin, as well as the provinces of Hebei, Shandong and Henan (see Methods section). During this period, the NCP region experienced the most severe O_3_ pollution nationwide, characterized by both higher O_3_ levels and widespread pollution areas (Fig. S1). Across the 37 cities, 1085 days were recorded on which the MDA8 O_3_ exceeded the national Grade II standard of 160 μg/m^3^, resulting in a regional average O_3_ non-attainment rate exceeding 30%. The annual evaluation concentrations of O_3_, calculated as the 90th percentile of the MDA8 O_3_, failed to meet national Grade II standards in 26 of the 37 cities. Notably, these cities are mainly situated in the central and western areas of the NCP, particularly at the junction of the Hebei, Henan and Shandong provinces (Fig. 1a). This spatial distribution exhibits a significant difference from the individual patterns observed for the O_3_ precursors, i.e. NO_2_ and VOCs (Fig. 1b and c). Moreover, the regions with elevated O_3_ levels align with areas experiencing higher temperatures and lower relative humidity (Fig. 1f, Fig. S2), which underscores the crucial influence of meteorological conditions on O_3_ production. Conversely, in the mountainous regions of northern Hebei Province and the coastal areas of eastern Shandong Province, the air quality was relatively good due to their advantageous geographical locations.

The severe O_3_ pollution in the NCP is primarily attributed to its high O_3_ production capacity. Utilizing an observation-based model (OBM; see Methods section), we estimated the *in situ* net O_3_ production rates (R_chem-O3_) across the 37 cities (Fig. 1e). The city-average rates (14–63 μg/m^3^/h) significantly exceeded those reported at urban locations in most regions in China, as well as at cities in North America, Europe and Japan, in the recent decade (Fig. S3). These high R_chem-O3_ values are sufficient to sustain the observed daytime O_3_ increase (R_obser-O3_) at urban sites (Fig. 1d and e). The spatial distribution of the O_3_ production rate mirrors the patterns of the O_3_ precursors, particularly NO_2_ (Fig. 1b), underscoring the pivotal role of NO_x_ in governing O_3_ formation. Notably, the local O_3_ formation in Beijing is much weaker than in the surrounding cities. This is attributed to the implementation of more stringent emission control measures in Beijing, which have more effectively reduced the precursor concentrations and consequently diminished its O_3_ production capacity [17,18].

**Figure 2.**
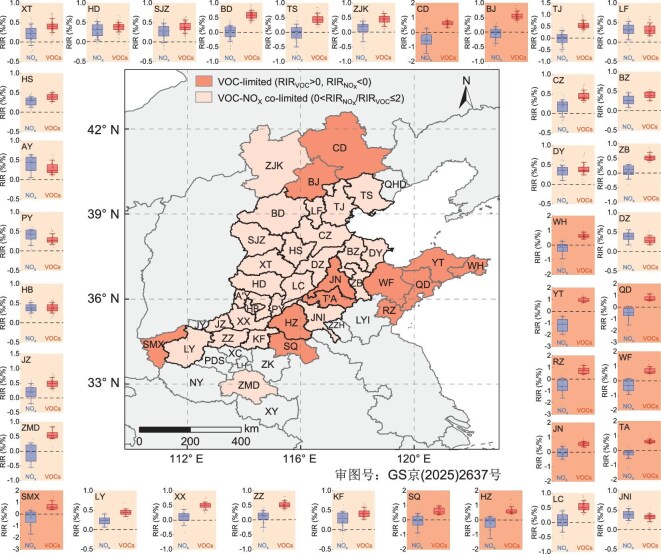
Spatial distribution of O_3_ formation regimes across 37 cities in the NCP region from 1 June to 31 August 2021. The daytime RIR values (07:00–19:00 local time) for the major O_3_ precursors (NO_2_ in blue box plot and VOCs in red box plot) in each city are distributed around the map. The box plots present the 10th, 25th, 50th, 75th and 90th percentiles of the daily RIR values, and the dots show the daily RIR values individually. The median RIR_NOx_/RIR_VOCs_ ratios for all cities are listed in Table S2, and the O_3_ formation regimes were determined as described in the Methods section. City colors on the map and undertones of the surrounding box plots denote VOC-limited (dark orange) and VOC–NO_x_ co-limited (light orange) regimes, respectively.

The O_3_ formation regime, examined through sensitivity simulations using the OBM (see Methods section), exhibits a distinct regional distribution across the NCP (Fig. 2). The majority of the cities (25 out of 37) fall within a VOC–NO_x_ co-limited regime. These cities are primarily clustered in the southern Hebei, western Shandong and central Henan provinces, which largely overlap with the regions experiencing the most severe O_3_ pollution (Fig. 1a). This coincidence aligns with the photochemical theory [e.g. the empirical kinetics modeling approach (EKMA) curve], which suggests that a VOC–NO_x_ co-limited regime corresponds to the highest potential for O_3_ production [19]. In contrast, the mountainous regions of northern Hebei Province and the coastal cities in eastern Shandong Province typically experience O_3_ formation under a VOC-limited regime. Analysis of the measurement data reveals that these cities have lower VOC concentrations and comparable NO_2_ levels, resulting in lower VOC/NO_2_ ratios (Fig. S4). Moreover, cities in the VOC-limited regime generally exhibit better air quality, and the reductions in NO_x_ emissions are relatively modest compared to those in other cities (Fig. S5).

A summary of previous research on the O_3_ formation sensitivity in key Chinese cities is documented in Table S3. Evidently, O_3_ formation in urban areas has primarily been VOC limited and NO_x_ saturated in the past decades. Consequently, the rise in O_3_ concentrations has accompanied the reduction of NO_x_ emissions in recent years [20,21]. This trend aligns with previous reports from the USA and Europe, where NO_x_ control led to elevated O_3_ levels from the 1990s to the 2010s [22–25]. Table S4 summarizes the changes in O_3_ formation regimes at a regional scale over the NCP in the past decade, based on results from air quality models and satellite observations. These studies indicated a substantial decline in VOC-limited regions, accompanied by an expansion of VOC–NO_x_ co-limited and NO_x_-limited regimes [13,20,26–29]. The shifts are particularly evident in the NCP compared with other regions, and are most pronounced in spring and summer [13,20,26–28]. However, these changes may largely reflect variations in the O_3_ formation regimes in rural areas [9,13]. Our investigation, grounded in synchronized field observations across 37 cities and uniform OBM analyses, reveals a transition in O_3_ formation from a VOC-limited regime to a VOC–NO_x_ co-limited regime in urban areas of the NCP, particularly in cities experiencing more serious O_3_ pollution. We further analyzed the multiyear variations in O_3_ formation regime in six major cities with available observations from multiple years (Text S1 and Fig. S6), as well as the long-term trends in satellite-retrieved HCHO/NO_2_ ratios (Text S2 and Fig. S7), both of which confirmed the shifting trend of O_3_ formation from a VOC-limited regime to a VOC–NO_x_ co-limited regime in this region. This transition can be attributed to the persistent efforts to control anthropogenic NO_x_ emissions in China over the past decade [30–32]. This transition mirrors the historical evolution observed in major US metropolitan areas, where sustained emission controls since the 1990s gradually shifted ozone formation from predominantly VOC-limited to increasingly NO_x_-limited regimes by the late 2000s to the early 2010s [33,34]. Therefore, we emphasize that urban O_3_ pollution control in the NCP region may have passed through the most challenging phase, where reducing NO_x_ emissions led to increased O_3_ concentrations. In the future, reducing either NO_x_ or VOC emissions will be effective in alleviating O_3_ pollution.

### Regional homogeneity of key O_3_ precursors, emission sources and control strategy

**Figure 3.**
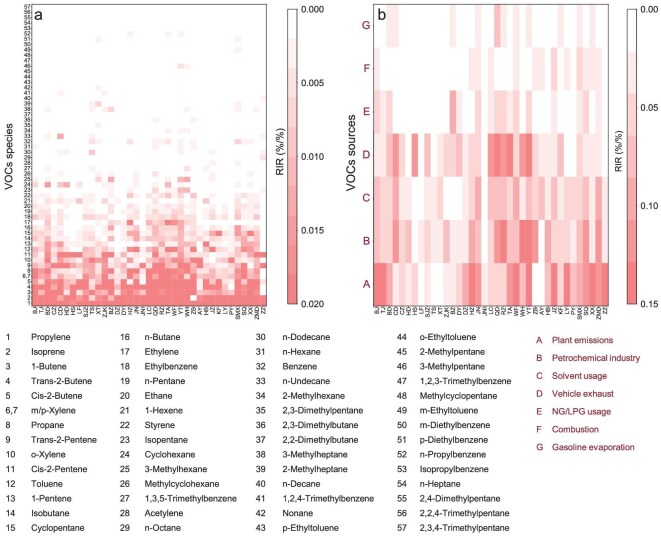
Regional distributions of major reactive VOC species and their emission sources across 37 cities in the NCP region. (a) Daytime average (07:00–19:00 local time) RIR values for the 57 VOC species (*m*-xylene and *p*-xylene cannot be separated in the GC–FID/MS and are treated as *m/p*-xylene), calculated using the OBM coupled with MCM v. 3.3.1. (b) The same as (a) except for the major VOC sources, which were identified by using the Positive Matrix Factorization model. Refer to Table S1 for the abbreviations of the cities.

The preceding analysis reveals that the formation of O_3_ in most cities in the NCP region has transitioned from a VOC-limited regime to a VOC–NO_x_ co-limited regime, where a sustained reduction in NO_x_ emissions is anticipated to yield satisfactory results. Concurrently, controlling the emissions of VOCs remains equally crucial, albeit more challenging due to their intricate compositions and significant variations in photochemical reactivity and emission source [35]. Herein, we delve into the regional disparities in the prevailing reactive VOC species across the 37 cities by individually computing the relative incremental reactivity (RIR) for 57 VOC species, using the OBM coupled with the comprehensive Master Chemical Mechanism (MCM v 3.3.1; see Methods section). Despite variations in the RIR values of individual VOCs across different cities, a general consistency is observed in the dominant reactive VOC species in most cities (Fig. 3a). These species include propylene, isoprene, 1-butene, *trans/cis*-2-butene, *m/p*-xylene, propane, *trans/cis*-2-pentene and *o*-xylene. The identification of these key VOCs aligns well with estimations of O_3_ formation potential (OFP) (Fig. S8a). These results highlight a clear regional homogeneity among the key VOC species, predominantly alkenes and aromatics, which contribute substantially to O_3_ formation in the NCP region. While previous studies have acknowledged the importance of alkenes and/or aromatic compounds in O_3_ production in northern China [8,21,36], our research advances this understanding by pinpointing specific reactive VOC species at a detailed level, based on concurrent comprehensive observations and advanced modeling in 37 cities. Additionally, biogenic VOCs, specifically isoprene, play a pivotal role in urban O_3_ formation, emerging as the top two most significant VOC species, both in terms of RIR and OFP, in 21 cities.

We conducted a standardized source apportionment analysis across the 37 cities to identify the primary emission sectors contributing to VOC concentrations and O_3_ formation (see Text S3 for detailed methods). Based on the analysis, the principal VOC sources include the petrochemical industry, solvent usage, vehicle exhaust, natural gas and liquefied petroleum gas usage (NG/LPG usage), combustion, gasoline evaporation, and plant emissions (Text S3 and Fig. S9). Subsequently, we calculated the source-specific RIR and OFP values to quantify the impact of each emission sector on O_3_ production. Notably, significant variations can be observed in the distribution of emission sectors among different cities. Nonetheless, the predominant VOC sources for O_3_ formation exhibit regional consistency across most cities (Fig. 3b and Fig. S8b). Specifically, plant emissions, petrochemical industry, solvent usage and vehicle exhaust constitute the primary sources of reactive VOCs contributing the most to RIR (81% ± 7%) and OFP (80% ± 8%), while the contributions from other sources are relatively minor. This indicates a clear regional homogeneity in the key VOC emission sources, highlighting potential targets for future VOC emission reduction strategies in the NCP region.

To devise effective strategies to control O_3_ pollution, we employed EKMA curves to determine the optimal VOCs/NO_x_ emission reduction ratio for the 37 cities (see Methods section and Text S4). Unexpectedly, most of the cities (35 out of 37) exhibit an optimal VOCs/NO_x_ reduction ratio within a narrow range of 1.8–3.7, with over 60% falling between 2.5 and 3.0 (Fig. S10). This finding contrasts significantly with previous reports in Chinese cities that demonstrated a large variability in the optimal VOCs/NO_x_ reduction ratios (Table S5). This discrepancy can be attributed to the transition of O_3_ formation from a VOC-limited to a VOC–NO_x_ co-limited regime. Under a VOC-limited regime, which was common during previous studies, O_3_ formation is highly sensitive to the magnitude of NO_x_ emission reduction. Specifically, slight NO_x_ reduction may lead to an increase in O_3_ levels, whereas a significant NO_x_ reduction would lower O_3_ levels. This sensitivity accounts for the large variability observed in the optimal VOCs/NO_x_ reduction ratios. However, under a co-limited regime, both NO_x_ and VOC emission reductions are effective in mitigating O_3_ levels, resulting in a more stable VOCs/NO_x_ reduction ratio. However, two oilfield cities, Dongying and Binzhou, present outliers with significantly higher optimal VOCs/NO_x_ reduction ratios of 5.7 and 6.0. These two cities are significantly influenced by extensive local activities related to oil extraction [37], which produce abundant VOC emissions. Hence, a much greater VOC reduction is deemed necessary in these regions to control O_3_ pollution effectively.

Overall, our findings demonstrate a remarkable consistency in the key reactive VOC species and their major emission sources, along with a narrow range of optimal VOCs/NO_x_ reduction ratios across most cities in the NCP region. This new observation, compared to the significant variability reported in the past, simplifies the implementation of regionally coordinated O_3_ pollution control strategies in the future. Based on our analysis, all cities can adopt similar strategies for O_3_ precursor reduction, which are anticipated to yield a synergistic regional effect owing to the homogeneity in O_3_ formation mechanisms and control policies.

### Future prospects for O_3_ pollution control in the NCP

Our observations and modeling analyses suggest that O_3_ formation in most urban areas of the NCP region has shifted from a primarily VOC-limited regime to a VOC–NO_x_ co-limited regime. This transition represents a crucial milestone in the effort to mitigate O_3_ pollution and highlights the potential efficacy of future reductions for NO_x_ and/or VOC emissions. In this section, we further evaluate the impacts of China’s long-term control policies on future O_3_ air quality.

**Figure 4.**
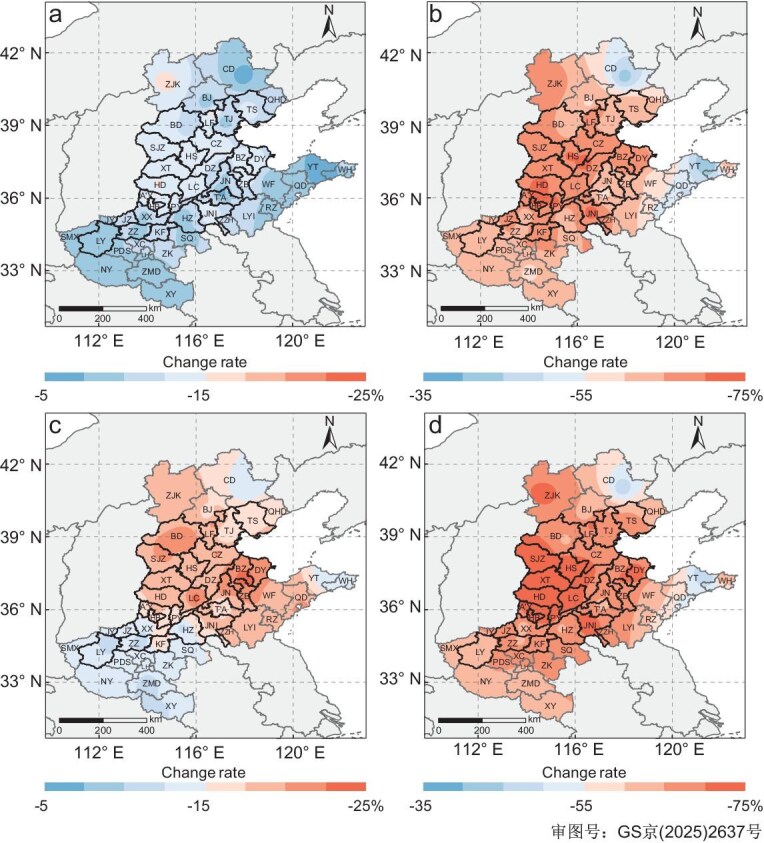
Model-predicted percentage reductions in MDA8 O_3_ concentrations across the 37 cities of the NCP region for 2030 and 2060. (a and b) Equal-proportional reduction approach: AVOC reductions applied proportionally across all species. (a) 2030: 22% NO_x_ and 26% AVOC reductions. (b) 2060: 88% NO_x_ and 61% AVOC reductions. (c and d) Source-allocated reduction approach: AVOC reductions allocated only to the three major anthropogenic sources (petrochemical industry, solvent usage, vehicle exhaust). (c) 2030: 22% NO_x_ and 26% AVOC reductions. (d) 2060: 88% NO_x_ and 61% AVOC reductions.

China’s most significant national policy is the ‘carbon neutrality’ objective, which involves stringent control measures to reach peak carbon dioxide emissions by 2030 and achieve carbon neutrality by 2060 [38]. This initiative is also expected to substantially reduce the emissions of various air pollutants, including NO_x_ and VOCs. According to the Dynamic Projection Model for Emissions in China (DPEC) [39], a robust combination of low-carbon policies and stringent enforcement of environmental regulations could reduce anthropogenic NO_x_ and anthropogenic VOC (AVOC) emissions in China by 22% and 26% by 2030, and by 88% and 61% by 2060, respectively, under the ‘double-carbon’ scenario, relative to 2020 levels. Utilizing the OBM model, we conducted a series of numerical simulations that involved the aforementioned reductions in NO_x_ and AVOC concentrations in each city using two approaches: the equal-proportional reduction approach and the source-specific reduction approach (see Methods section and Text S4). By 2030, the control policy is projected to reduce the annual evaluation concentrations of O_3_ by 11.2% ± 3.6% (range: 2.8% to 16.2%) across the 37 cities under the equal-proportional reduction approach (Fig. 4a), whereas the source-specific reduction approach yields a larger decrease of 17.2% ± 4.0% (range: 9.3% to 26.5%) (Fig. 4c). The simulated O_3_ reduction ratios are close to those (7%–13%) obtained from chemical transport model simulations [40]. By 2060, more substantial decreases in O_3_ levels are anticipated, with an average reduction of 63.4% ± 8.4% (range: 36.5% to 73.3%) under the equal-proportional reduction approach (Fig. 4b) and 66.1% ± 7.4% (range: 42.5% to 74.4%) under the source-specific reduction approach (Fig. 4d). These results demonstrated that O_3_ pollution can be more effectively mitigated when VOC emission reductions are targeted toward the major anthropogenic sources rather than applied uniformly across all VOC sources. Moreover, the greater projected reduction in O_3_ during the latter period (2030–60) can be attributed to the shift in O_3_ formation from a VOC–NO_x_ co-limited to an NO_x_-limited regime. This transition to a low-NO_x_ condition is considered the optimal strategy for mitigating O_3_ pollution [16]. Indeed, by 2060, all cities are expected to be under an NO_x_-limited regime for O_3_ formation (Fig. S11). These results indicate that significant improvements in O_3_ air quality can be expected with the progress of China’s ‘carbon neutrality’ initiative. Note that we consider only future emission reductions, which are approximated as proportional decreases in ambient concentrations in the OBM simulations. We acknowledge that neglecting meteorological variability may introduce uncertainties into the projected O_3_ changes. Nevertheless, historical data showed that relative change in anthropogenic emissions and observed atmospheric concentrations have been highly consistent over the past decade (Table S6), indicating that this assumption is reasonable and appropriate for our study region.

In this study, we also observed significant contributions of biogenic VOCs (BVOCs) to O_3_ formation in urban areas across the NCP. This finding aligns with recent reports highlighting the growing contribution of BVOC emissions from urban landscapes and heatwaves to urban O_3_ pollution [41–44]. The influence of BVOCs on O_3_ formation is anticipated to intensify with the ongoing reduction in AVOCs and NO_x_ emissions. This is substantiated by the O_3_–precursor relationships projected for 2060, which indicate that BVOCs will surpass AVOCs and contribute more to O_3_ formation, as indicated by their higher RIR values (Fig. S11). Previous research has suggested that BVOC emissions in China could increase by approximately 10%–60% by 2060 in the context of climate change [45–47]. In this study, we assessed the potential impact of increased BVOC emissions on future O_3_ pollution and determined that a 60% rise in BVOC emissions could elevate MDA8 O_3_ by up to 6.8% in the NCP in 2060 (Fig. S12). However, the impact of BVOC emissions on future O_3_ pollution will not be as large in 2060, when O_3_ formation will be dominantly limited by the availability of NO_x_.

### Policy implications

Effective urban O_3_ pollution control generally entails two critical transition stages: from a VOC-limited to a VOC–NO_x_ co-limited regime, and eventually to an NO_x_-limited regime. Although previous studies have suggested the potential ineffectiveness of China’s stringent air pollution control measures in addressing O_3_ pollution, this study offers a new perspective by integrating extensive field observations and comprehensive model simulations. It reveals the remarkable effectiveness of these control efforts in the heavily polluted NCP region. While the measures have not directly reduced ambient O_3_ concentrations, they have significantly altered the O_3_ formation mechanism. This shift has occurred from a primarily VOC-limited (NO_x_ saturation) regime to a VOC–NO_x_ co-limited regime in most cities or is underway in the remaining cities. Identifying this transition is crucial for informing the next phase of O_3_ control strategies and strengthening policy confidence. Furthermore, our analysis uncovers remarkable regional homogeneity in the primary reactive VOC species, their major sources, and the optimal VOCs/NO_x_ emission reduction ratios across urban areas within the NCP. This homogeneity presents promising opportunities for enhancing regionally coordinated control strategies. In the future, targeting VOC emission reductions from major anthropogenic sources, such as the petrochemical industry, solvent usage and vehicle exhaust, will be more effective in mitigating O_3_ pollution than applying a uniform reduction strategy. These findings underscore the significant progress achieved through China’s O_3_ pollution control efforts, which are approaching a critical juncture. This progress suggests greater flexibility and feasibility in devising NO_x_ and VOC emission reduction strategies, as well as in implementing regionally coordinated control measures. Therefore, an optimistic perspective can be maintained for O_3_ pollution control in the future.

## METHODS

### Field observations

The comprehensive field measurement campaign was a part of the Integrated Field Atmospheric Composition Test for complex air pollution in North and East China in 2021 (IFACT2021-N&E China), spanned from 1 June to 31 August [48]. The campaign concurrently measured trace gases and aerosols across 88 major cities in 10 provinces and municipalities of North and East China. Notably, 37 cities in the heavily polluted NCP underwent detailed real-time measurements of VOCs. To our knowledge, this constituted the largest intensive field measurement campaign specifically aimed at tackling regional-scale photochemical air pollution in China.

The concentrations of six criteria air pollutants (O_3_, NO_2_, CO, SO_2_, PM_2.5_ and PM_10_) and meteorological parameters (temperature, relative humidity, pressure, wind speed and wind direction) were routinely monitored using commercial instruments by the China National Environmental Monitoring Center (CNEMC). Fifty-seven VOCs, including 29 alkanes, 10 alkenes, 1 alkyne and 17 aromatics were detected using commercial gas chromatography−flame ionization detector/mass spectrometers (GC-FID/MS) at an hourly resolution in 37 cities. To ensure cross-site comparability among the GC-FID/MS systems deployed across cities, all instruments followed a unified standard operating procedure (SOP; https://www.cnemc.cn/jcgf/dqhj/202109/t20210924_953138.shtml). Strict quality assurance and quality control procedures were consistently implemented in accordance with the technical guidelines issued by the CNEMC.

### OBM simulations

Chemical box models were employed to simulate the photochemical production of O_3_ and to assess the O_3_–precursors relationships. The model incorporated two widely used atmospheric chemistry mechanisms: Regional Atmospheric Chemistry Mechanism version 2 (RACM2) [49] and MCM version 3.3.1 [50]. We compared their performances in simulating O_3_ production rates and found a high degree of concordance for all 37 cities (Fig. S13). To optimize computational resources, we predominantly used the RACM2 model, while the MCM v3.3.1 model was employed to calculate the RIR values for the individual VOC compounds, enabling species-level RIR computations, across the 37 cities in the NCP region. Detailed information on the model settings is provided in Text S4.

O_3_ production rates and RIR were calculated [51,52]. Based on the ratio of RIR values for NO_x_ and VOCs, the O_3_ formation regimes were classified into three types: VOC-limited; NO_x_-limited; and VOC–NO_x_ co-limited regimes. In this study, the VOC-limited regime is characterized by a positive RIR for VOCs and a negative RIR for NO_x_. The NO_x_-limited regime is defined by positive RIRs for both NO_x_ and VOCs, with the RIR for NO_x_ being at least twice that of VOCs. The VOC–NO_x_ co-limited regime is identified when both RIRs for NO_x_ and VOCs are positive and comparable, with ratios of RIR_NOx_/RIR_VOCs_ ranging from 0 to 2 [53,54]. The EKMA approach was employed to determine the optimal AVOCs/NO_x_ reduction ratio for the 37 cities [14]. Detailed calculation methods for O_3_ production rates, RIR and EKMA are provided in Text S4.

Scenarios of national control policy were conducted using two approaches: (i) the equal-proportional reduction approach, in which the projected VOC reductions were uniformly applied across all VOC species (Fig. 4a and b); and (ii) the source-specific reduction approach, in which the projected VOC reductions were allocated among three major anthropogenic sources: petrochemical industry; solvent usage; and vehicle exhaust (Fig. 4c and d). The impact of climate change was assessed through sensitivity analyses, which showed that the emission-reduction strategy under the ‘double-carbon’ scenario continues to effectively mitigate O_3_ pollution under future meteorological conditions, albeit with a slightly reduced magnitude of O_3_ decline (see Text S4 for detailed evaluation).

## Supplementary Material

nwaf596_Supplemental_File
