# Supplementary material for: Critical transition of urban ozone formation regime in the North China Plain
Source: Natl Sci Rev. 2026 Jan 2;13(3):nwaf596. doi: 10.1093/nsr/nwaf596 (PMC12878323; doi:10.1093/nsr/nwaf596)
Supplement: nwaf596_Supplemental_File [file nwaf596_supplemental_file.pdf]

Supplementary for

Critical transition of urban ozone formation regime in the North China Plain

Likun Xue[1]*, Yujiao Zhu[1]*, Jian Gao[2]*, Xuelian Zhong[1], Can Cui[1], Shuai Wang[3], Zhiwen Jiang[4], Yue Sun[1], Qinyi Li[1], Yuqiang Zhang[1], Hong Li[2], Yingnan Zhang[1], Shanshan Wang[4], Min Zhao[1], Hengqing Shen[1], Yujie Zhang[2], Guigang Tang[3], Tao Wang[5], Wenxing Wang[1,2]

[1]Environment Research Institute, Shandong University, Qingdao 266237, China.

[2]State Key Laboratory of Environmental Criteria and Risk Assessment, Chinese Research Academy of Environmental Sciences, Beijing 100012, China.

[3]China National Environmental Monitoring Centre, Beijing 100012, China.

[4]Shanghai Key Laboratory of Atmospheric Particle Pollution and Prevention (LAP[3]), Department of Environmental Science and Engineering, Fudan University, Shanghai 200438, China.

[5]Department of Civil and Environmental Engineering, The Hong Kong Polytechnic University, Hong Kong 999077, China.

*Corresponding authors: Likun Xue (xuelikun@sdu.edu.cn), Yujiao Zhu (zhuyujiao@sdu.edu.cn) and Jian Gao (gaojian@craes.org.cn)

This file includes:

Text S1 to S4

Fig. S1 to S15

Table S1 to S6

SI References

Text S1. Multi-year variations in O_3 formation regimes based on multiple years observations and OBM simulations.

Six major cities (i.e., BJ, TJ, JN, ZZ, SJZ and DY) were selected to analyze multi-year variations in O_3 formation regimes with available multiple years observations. As shown in Fig. S6, obvious increasing trends in RIR for NO_x were observed in BJ (2019-2021), JN (2020-2022), ZZ (2019-2021), SJZ (2020-2021) and DY (2022-2023), indicating a growing sensitivity of O_3 formation to NO_x in these cities. Notably, a clear turning point occurred in ZZ and SJZ, where O_3 formation regime shifted from VOC-limited in 2020 to VOC-NO_x co-limited in 2021. DY had already entered the VOC-NO_x co-limited regime before 2021, and the RIR for NO_x continued to increase thereafter. In the remaining cities, the median RIR values for NO_x in 2021 were -0.009 in BJ, -0.05 in TJ and -0.13 in JN, suggesting that these cities were on the verge of transitioning to a VOC-NO_x co-limited regime. Overall, the available multi-year observations and OBM simulations confirmed the marked increase in RIR for NO_x, indicating that the O_3 formation regime has either already entered or is in the process of shifting toward a VOC-NO_x co-limited regime.

Text S2. Long-term trends in satellite-retrieved ground-level (0-100 m) $HCHO/NO_2$ ratios.

We analyzed the long-term variations in the satellite-retrieved $HCHO/NO_2$ ratios using data from OMI (2014-2019) and TROPOMI (2019-2022) for five major cities (i.e.,BJ, TJ, JN, ZZ and SJZ). Note that 1) only grid cells located within urban areas of each city were included, while the suburban and rural regions were excluded from the analysis; and 2) the 3D spatial distribution of $HCHO/NO_2$ ratios were reconstructed, with only ground-level (0-100 m) $HCHO/NO_2$ ratios were considered in this study [1]. As shown in Fig. S7, the ground-level $HCHO/NO_2$ ratios exhibited a clear upward trend in these cities, further indicating the increasing sensitivity of O_3 formation to NO_x.

Text S3. Source apportionment analysis.

The US Positive Matrix Factorization (PMF) model, version 5.0, was employed to apportion sources of the measured VOCs across 37 cities [2]. PMF assumes that the measured concentrations of VOC species at the receptor sites are the result of linear combinations of contributions from various sources [3]. Specifically, this methodology allows for the decomposition of the input data matrix X, with dimensions $i \times j$, into two matrices with

distinct interpretations: the source contribution matrix (*G*) and the pollutant profiles matrix (*F*), as illustrated in the following Equation (1).

$$X = G \times F \quad (1)$$

The concentrations of 57 VOCs and their associated uncertainties were employed as input parameters for the model. The input uncertainty was quantified using Equations (2) and (3), depending on whether the measured concentrations were below or above the method detection limit (MDL). Equation (2) was applied when the concentrations did not exceed the MDL, whereas Equation (3) was used for concentrations surpassing this limit.

$$uncertainly = \frac{5}{6} \times MDL \quad (2)$$

$$uncertainty = \sqrt{(EF \times concentration)^2 + (0.5 \times MDL)^2} \quad (3)$$

where EF represents error fraction, which was set to 20% in this study.

Building upon the input files, the model employs the least squares method to minimize the Q value for estimating the composition and contribution of pollution sources. The quality of the input data was quantified in terms of the signal-to-noise ratio (S/N) as indicated by the model. Species with an S/N below 0.5 were categorized as "bad" and were subsequently excluded from the PMF analysis. Those with an S/N between 0.5 and 1.0 were classified as "weak", whereas compounds with an S/N above 1.0 were deemed "strong" [2]. The determination of the optimal factor number solution is based on two criteria: (1) the ratio of Q to $Q_{expected}$ and (2) the physical plausibility of the factors [4].

In this study, seven major sources were identified from the PMF for the 37 cities: vehicle exhaust, solvent usage, petrochemical industry, gasoline evaporation, natural gas and liquefied petroleum gas usage (NG/LPG usage), combustion, and plant emissions (Fig. S9).

Vehicle exhaust contains moderate proportions of light alkanes (e.g., ethane, propane, and butanes), and aromatics (e.g., benzene, toluene, and 1, 2, 4-trimethylbenzene, etc.), and high proportions of heavy alkanes (e.g., nonane, *n*-decane, and *n*-dodecane). They also represent a significant source of ethylene and propylene, with an ethylene-to-acetylene ratio and a propylene-to-acetylene ratio of 1.03 and 1.41, respectively, which is consistent with typical traffic exhaust emissions [5].

Solvent usage involves high percentages of aromatics, including benzene, toluene, ethylbenzene, xylenes, and 1, 2, 4-trimethylbenzene. These species constitute mainly fugitive emissions produced by the evaporation of solvents during painting applications, printing processes, and the production of synthetic spices, adhesives, and cleaning agents [6].

Petrochemical industry is mainly involved in refining and processing petrochemical products, characterized by high concentrations of reactive alkenes such as ethylene, propylene, and butenes. These compounds are emitted during refining and alkene production, as well as throughout storage and loading operations [7-8]. Styrene, a significant chemical product monomer, also constitutes a high proportion of this source [9].

Gasoline evaporation encompasses VOC emissions produced during the production, usage, storage, and delivery of gasoline, characterized by high proportions of isopentane and n-pentane [10].

NG/LPG usage represents a combination of natural gas usage and liquefied petroleum gas usage. Butane is the main component of liquefied petroleum gas, while ethane and propane are the main component of natural gas [11].

Combustion is distinguished by high percentages of acetylene, as well as high levels of ethylene, benzene, etc. [11,12].

Plant emissions include large fractions of isoprene, a tracer indicative of biogenic sources [13].

Text S4. Observation-based model (OBM) settings and computations

Observation-based model incorporated two widely-used atmospheric chemistry mechanisms: Regional Atmospheric Chemistry Mechanism version 2 (RACM2) and Master Chemical Mechanism version 3.3.1 (MCM). RACM2 is a condensed gas-phase mechanism that accounts for 363 reactions among 21 inorganic and 98 lumped organic species [14]. MCM v3.3.1 is an explicit gas-phase mechanism that details a comprehensive set of more than 17000 reactions involving 5800 individual organic species and the latest inorganic chemistry reactions [15]. The efficacy of these mechanisms in simulating O_3 production and atmospheric photochemistry has been substantiated by prior research [16-17].

The model incorporates key physical processes, including dry deposition and dilution mixing,

by integrating the diurnal variations of the boundary layer height (BLH). The BLH is assumed to rise from 300 m at dawn to a peak of 1500 m at 14:00 local time, and remain constant until dusk, and then decreases back to 300 m following previous study [18]. Sensitivity analyses with alternative maximum mixing heights (e.g., 500, 1000 m and 2000 m) revealed limited impact on the model outcomes, with net O_3 production rates varying by less than 5%. Ground-based solar radiation measurements were not available in this study; therefore, photolysis frequencies were computed as a function of the solar zenith angle under the assumption of clear sky conditions [19]. Sensitivity analyses indicated that cloudy conditions (e.g., all photolysis rates decreased by 20 %) resulted in a moderate decrease in the net O_3 production rates (~ 17%), while exerting limited effects on the calculated RIR values and no changes in O_3 formation regimes. The dilution effect was accounted for by employing a dilution mixing rate that varies with the BLH. Hourly observations of O_3, NO_2, CO, SO_2, VOCs, temperature, and relative humidity were input into the model, with missing data linearly interpolated to maintain a 1-hour resolution. Note that the modelling results with >20% observational data gap were discarded from the formal analysis. NO concentrations were reconstructed using a photostationary-state (PSS) approach constrained by observed NO_2, O_3, and j(NO_2). The OBM simulations reproduce the observed NO reasonably well, and the resulting RIR values remain robust (Fig. S14). Oxygenated VOCs (OVOCs) were not measured, and we evaluated the OBM-simulated OVOCs; the model performs reasonably well, and the RIR values remain robust (Fig. S15). Heterogeneous HO_2 uptake on aerosols were included, with an uptake coefficient (γ_{HO2}) set to 0.08 [20]. HONO concentrations were constrained using HONO/NO_2 ratios of 0.04 during the daytime and 0.05 at nighttime [21]. A two-day pre-run was conducted to establish a steady state for unconstrained species (e.g., OH radical) within the model. This model has been applied in many previous studies and further details can be found elsewhere [18, 22, 23].

Computation of O_3 production rates: the model has an automated procedure to calculate the production and loss rates of O_3, incorporating observational data constraints and chemical mechanisms. Specifically, the O_3 production rate is determined by summing the reaction rates of HO_2+NO and RO_2+NO; the O_3 loss rate is computed based on the reactions involving O_3 photolysis, O_3+OH, O_3+HO_2, O_3+VOCs, NO_2+OH, NO_2+RO_2, as well as the heterogeneous reactions of NO_3 and N_2O_5 [18]. The net O_3 production rate is then derived by subtracting the O_3 loss rate from the O_3 production rate.

Calculation of Relative Incremental Reactivity (RIR): The RIR is the most widely utilized metric for assessing the sensitivity of O_3 production to its precursors. It is defined as the ratio of the percentage decrease in the net O_3 production rate to the percentage reduction in the concentration (or emissions) of a specific O_3 precursor [24]. This metric can be determined through OBM sensitivity modeling simulations, which involve reducing the concentrations (by 20% in this study) of specific O_3 precursors, such as NO_x and VOCs, including their various sub-groups or species. In the current study, a large number of modeling simulations were conducted to compute the RIR values for the principal O_3 precursors and their emission sources. Specifically, the daytime average (07:00–19:00 local time) RIRs for major O_3 precursors, including NO_x, AVOCs, and BVOCs, as well as the primary AVOC groups (i.e., alkanes, alkenes, and aromatics), were calculated daily from June 1 to August 31, 2021, across 37 cities. Secondly, campaign-average RIR values for 57 individual VOC species were derived for the 37 cities using the explicit MCM v3.3.1 model. Lastly, RIR values for the major VOC emission sources were also determined for all 37 cities, by integrating the PMF-identified VOC sources into the OBM model.

Empirical kinetic modeling approach (EKMA) simulations.

The EKMA was employed to ascertain the optimal AVOCs/NO_x reduction ratio for the 37 cities included in this study. The average conditions throughout the observation period, from June 1 to August 31, 2021, were considered as the baseline for the initial box model inputs. A total of 441 modeling scenarios were generated by adjusting NO_x and AVOC levels relative to the baseline (21 NO_x levels and 21 AVOC levels were set, with reductions to a minimum of 0 and increases to a maximum of 1, incremented at 5% intervals of their average values). The EKMA plot was made based on the MDA8 O_3 simulated by each scenario. Note that BVOCs were not included in the EKMA analysis, and the observed diurnal variations of isoprene concentrations were input into the OBM to maintain the reasonable chemical environment in the model. In the EKMA plot, the ridge line was established by identifying the maxima of each isopleth. The AVOCs/NO_x ratio along this ridge line signifies the optimal reduction ratio for controlling AVOCs and NO_x concentrations, which is defined as the most efficient AVOCs/NO_x ratio for O_3 pollution mitigation [25].

Scenarios of national control policy: For each city, we used the average mixing ratios of O_3 precursors (NO_x and VOCs) between June 1 and August 31, 2021 as the baseline scenario.

According to the emission reduction targets of "Dynamic Projection Model for Emissions in China", NO_x and VOCs concentrations were reduced by 22% and 26% for the 2030 scenario and by 80% and 61% for the 2060 scenario, respectively. The corresponding RIR values and MDA8 O_3 concentrations were then simulated using two approaches: (1) the equal-proportional reduction approach, in which the projected VOC reductions were uniformly applied across all VOC species (Fig. 4a-b), and (2) the source-specific reduction approach, in which the projected VOC reductions were allocated among three major anthropogenic sources: petrochemical industry, solvent usage, and vehicle exhaust (Fig. 4c-d). The impact of climate change was assessed through sensitivity analyses as follows:

Future meteorological conditions (temperature, radiation, and relative humidity) were taken from the Coupled Model Intercomparison Project Phase 6 (CMIP6) consistent with the Shared Socioeconomic Pathway (SSP) scenarios used in the DPEC framework. Two representative cities were selected: JN representing the VOC-limited regime and TJ representing the VOC–NOx co-limited regime.

For JN, the projected 2030 meteorological conditions (averaged over 2028-2032) relative to 2021 (averaged over 2020-2022) show increases of 3.1% in temperature and 1.7% in radiation, along with a decrease of 3.4% in relative humidity. By 2060 (averaged over 2058-2062), these changes are projected to reach +11.9% for temperature, +4.5% for radiation, and -18.1% for relative humidity. Using 2021 observations as a baseline, we scaled temperature, radiation, and relative humidity according to these projected ratios to represent future meteorological conditions. The adjusted meteorological parameters were then used as OBM inputs to recalculate future O_3 concentrations. The results showed that incorporating future meteorology, O_3 concentration deceased by 6.40% in 2030 and 57.22% in 2060, compared with decreases of 9.45% and 59.64%, respectively, when future meteorology was not considered.

For TJ, projected 2030 meteorological conditions show increases of 3.7% in temperature and 2.2% in radiation, along with a decrease of 5.5% in relative humidity. By 2060, these changes are projected to reach +9.2% for temperature, +4.8% for radiation, and -11.6% for relative humidity. Applying the same scaling approach, the adjusted meteorology yields O_3 concentration deceased by 4.93% in 2030 and 59.2% in 2060, compared with decreases of 8.04% and 61.53% under fixed meteorology.

Similar analyses were performed for other cities. Overall, the results show that the emission-reduction strategy under the "double-carbon" scenario continues to effectively mitigate O_3 pollution under future meteorological conditions, albeit with a slightly reduced magnitude of O_3 decline.

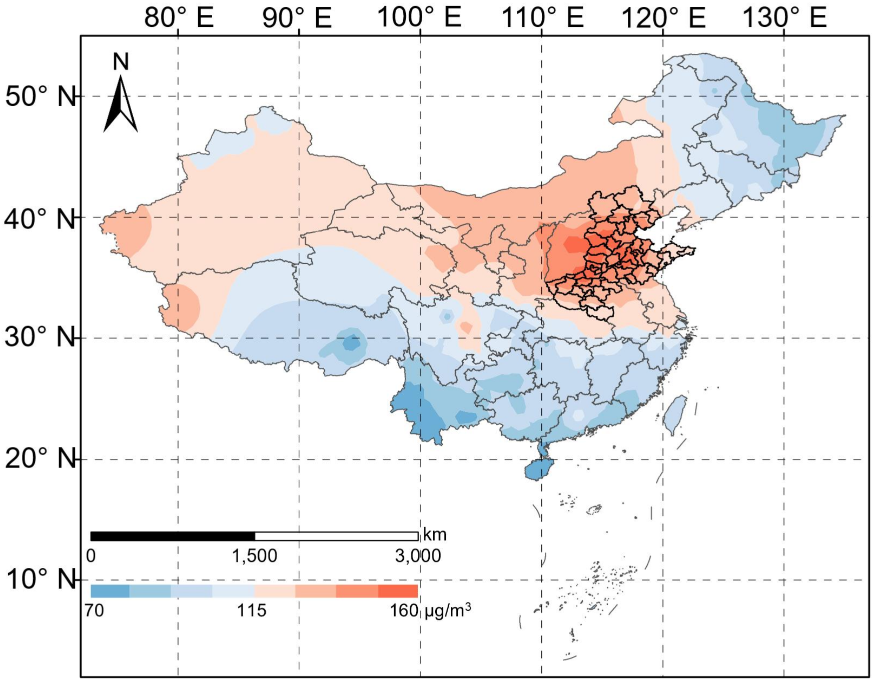

Fig. S1. Spatial distributions of the 90th percentile of the maximum daily 8-hour average concentration of O_3 (MDA8 O_3-90th) over China during June 1 and August 31, 2021. Data sourced from China National Environmental Monitoring Center (CNEMC). The areas with solid black borders are the target cities in this study.

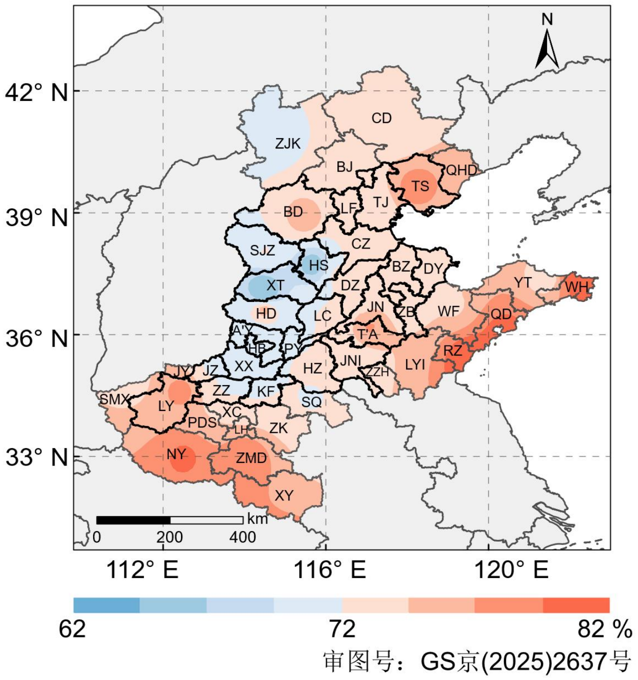

Fig. S2. Spatial distributions of the relative humidity (RH) in the NCP region during June 1 - August 31, 2021. The plot is created based on the spatial interpolation of the data in each city. Areas with solid black borders denote the 26 cities that failed to meet the national Grade II standard (160 μg/m^{-3} for MDA8 O_3). Refer to Table S1 for the abbreviations of the cities.

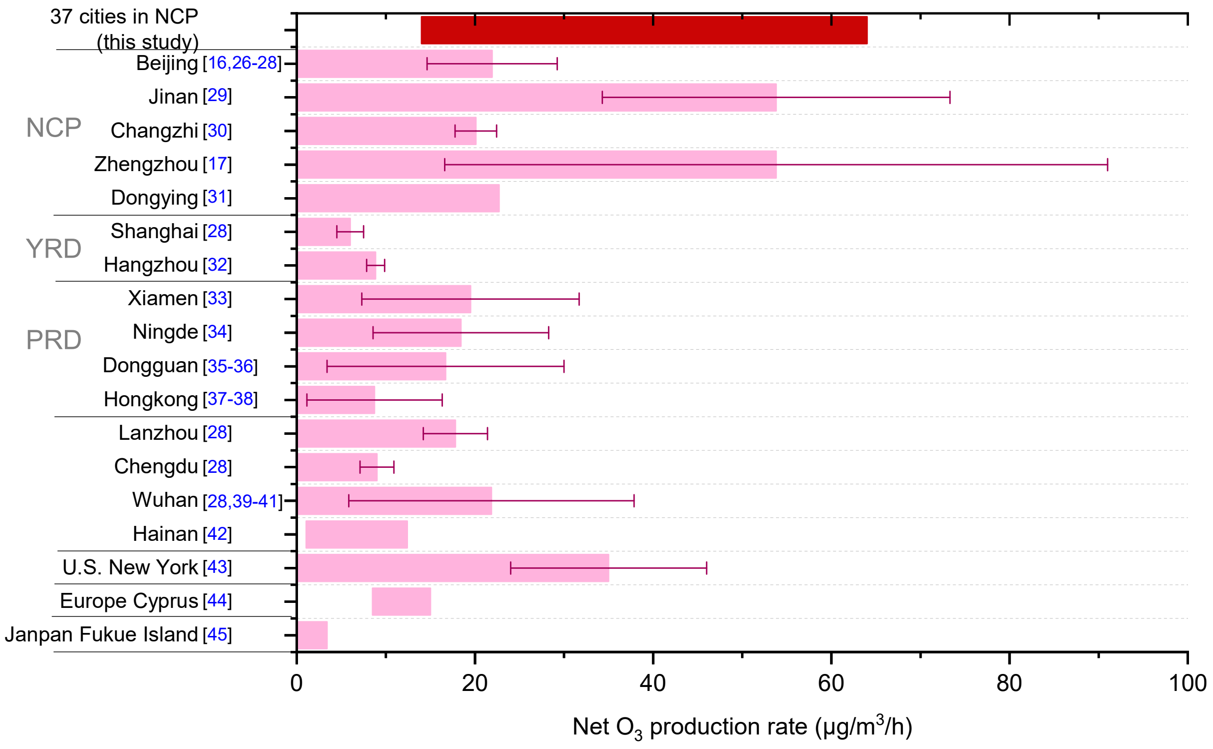

Fig. S3. In-situ net O_3 production rates in various cities from previous studies and this study, calculated using observation-based models (OBM). The literature review was conducted using "Web of Science" with the keywords "O_3 production rate" and "observation based model" and was restricted to publications from 2010 to 2025. From the 85 relevant references identified, 22 papers reporting O_3 production rates at urban sites were selected. In this figure, the red column represents the range of values across the 37 cities in the NCP from this study. The pink columns with error bars show the mean and standard deviation calculated based on the data reported in the literature, while the pink columns without error bars represent the ranges of the values. References are shown alongside the corresponding city names.

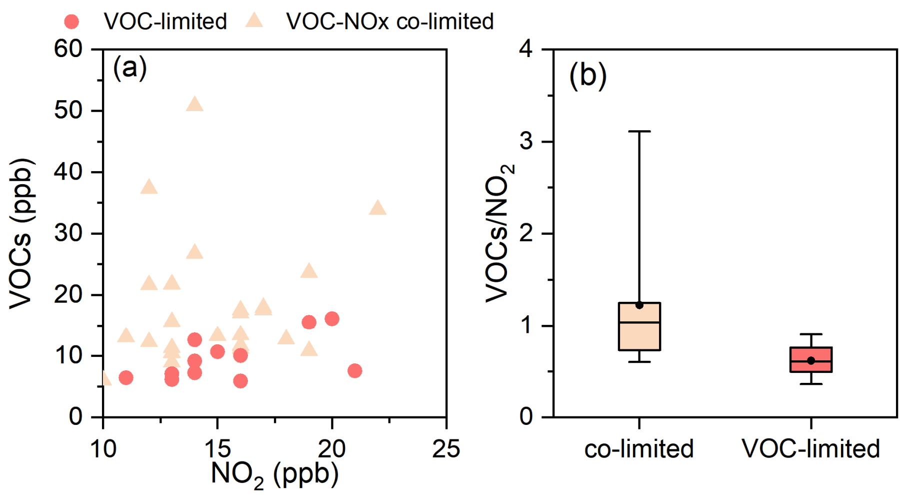

Fig. S4. (a) Scatter plot of VOCs and NO_2 concentrations in 37 cities. (b) Box plot of VOC/NO_2 ratios under VOC-limited and VOC–NO_x co-limited regimes. The box plots display the 5th, 25th, 50th, 75th, and 95th percentiles of the VOCs/NO_2 values.

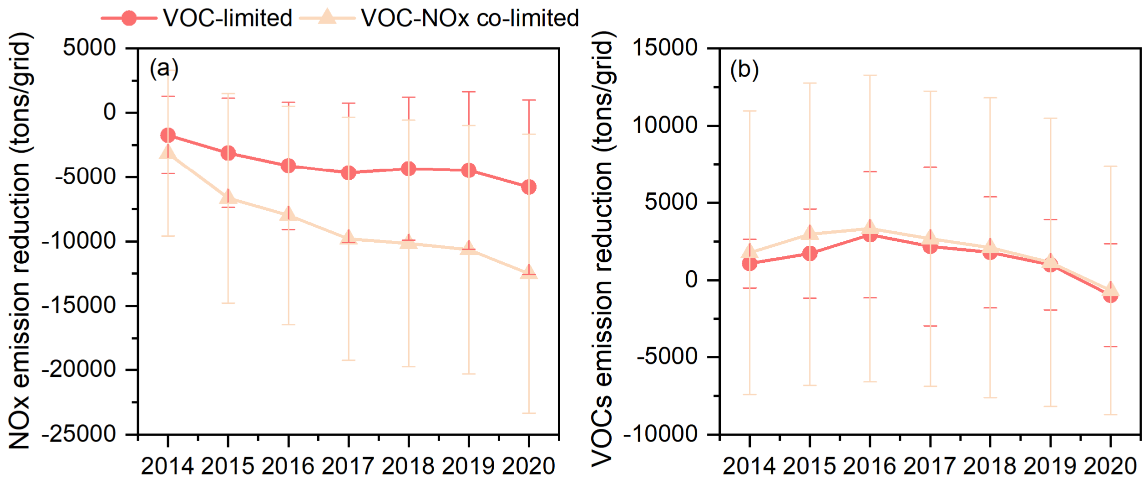

Fig. S5. Averaged annual emission reductions of NOx and VOC in each city relative to 2013, under VOC-limited and VOC–NO_x co-limited regimes. Emission data were obtained from the Multi-resolution Emission Inventory for China (MEIC, http://www.meicmodel.org). The spatial resolution for each city was 0.25° × 0.25°.

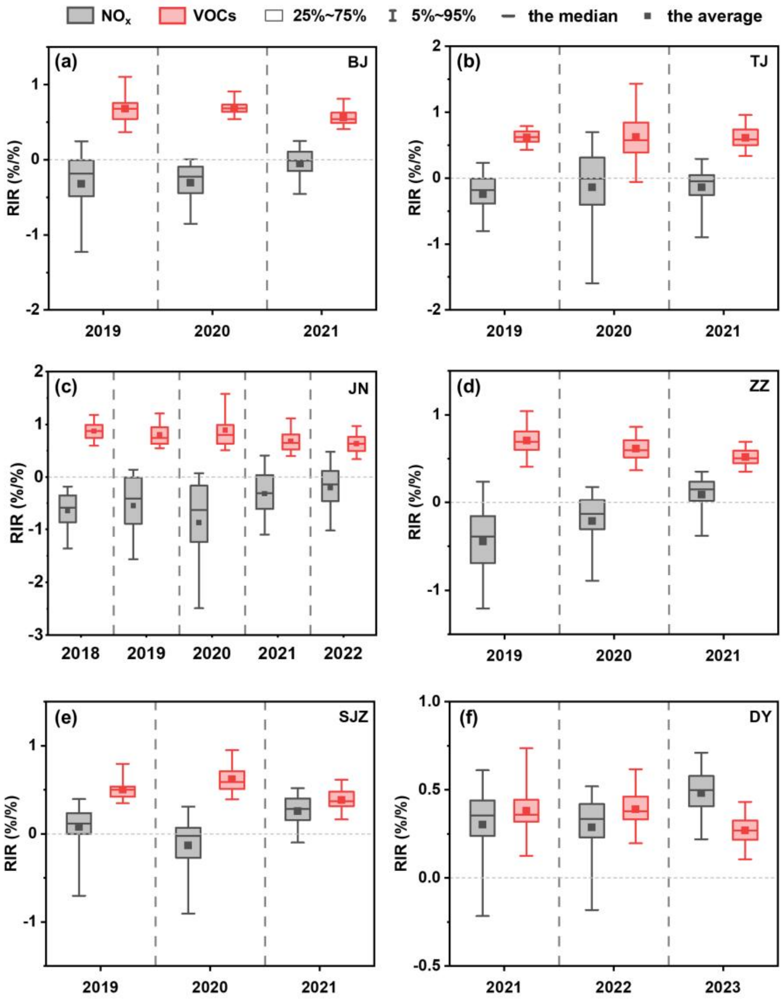

Fig. S6. Daytime RIR values (07:00–19:00 local time) for the major O_3 precursors, NO_x (gray box plot) and VOCs (red box plot) in representative cities: BJ, TJ, JN, ZZ, SJZ, and DY, during June to August across multiple years. The box plots display the 5th, 25th, 50th, 75th, and 95th percentiles of the daytime RIR values.

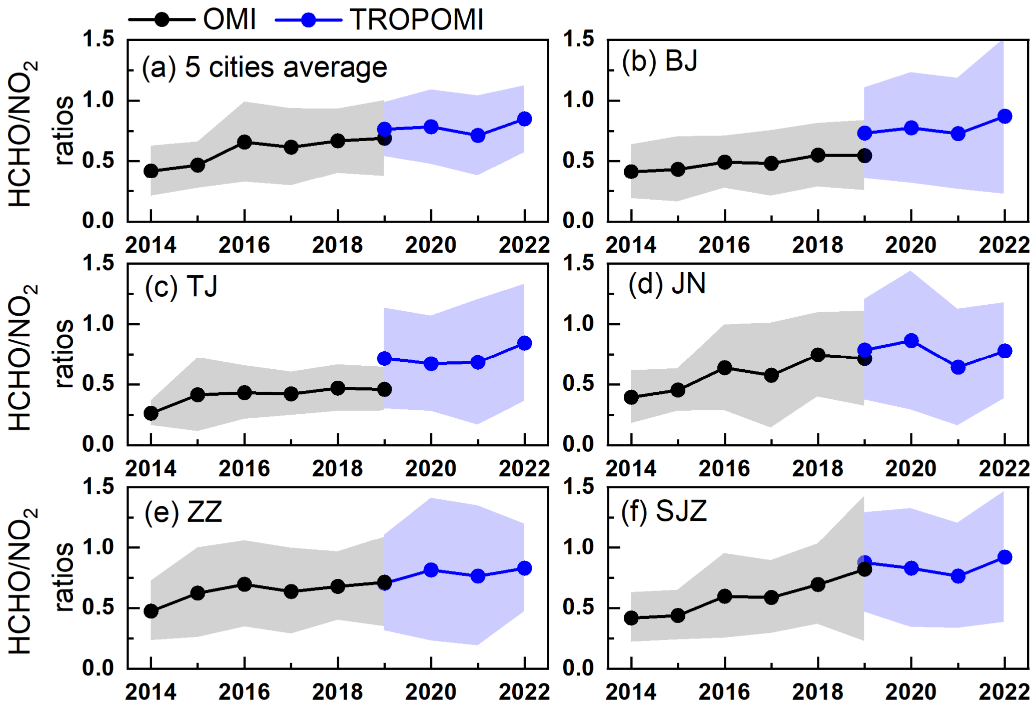

Fig. S7. Long-term trends in the satellite-retrieved HCHO/NO_2 ratios at ground-level (0-100 m) in BJ, TJ, JN, ZZ, and SJZ, as well as the average across the five cities, during June–August from 2014 to 2022. Tropospheric column HCHO and NO_2 data were obtained from the OMI/Aura Level 3 daily global product (2014–2019, accessed on 5 July 2024, black dots) and the TROPOMI Level 3 daily global product (2019–2022, accessed on 25 January 2025, blue dots). OMI and TROPOMI showed strong consistency during the overlapping year (2019). The method for reconstructing 3D spatial distribution of FNR follows Jiang et al. (2025) [1]. Shaded areas indicate standard deviations.

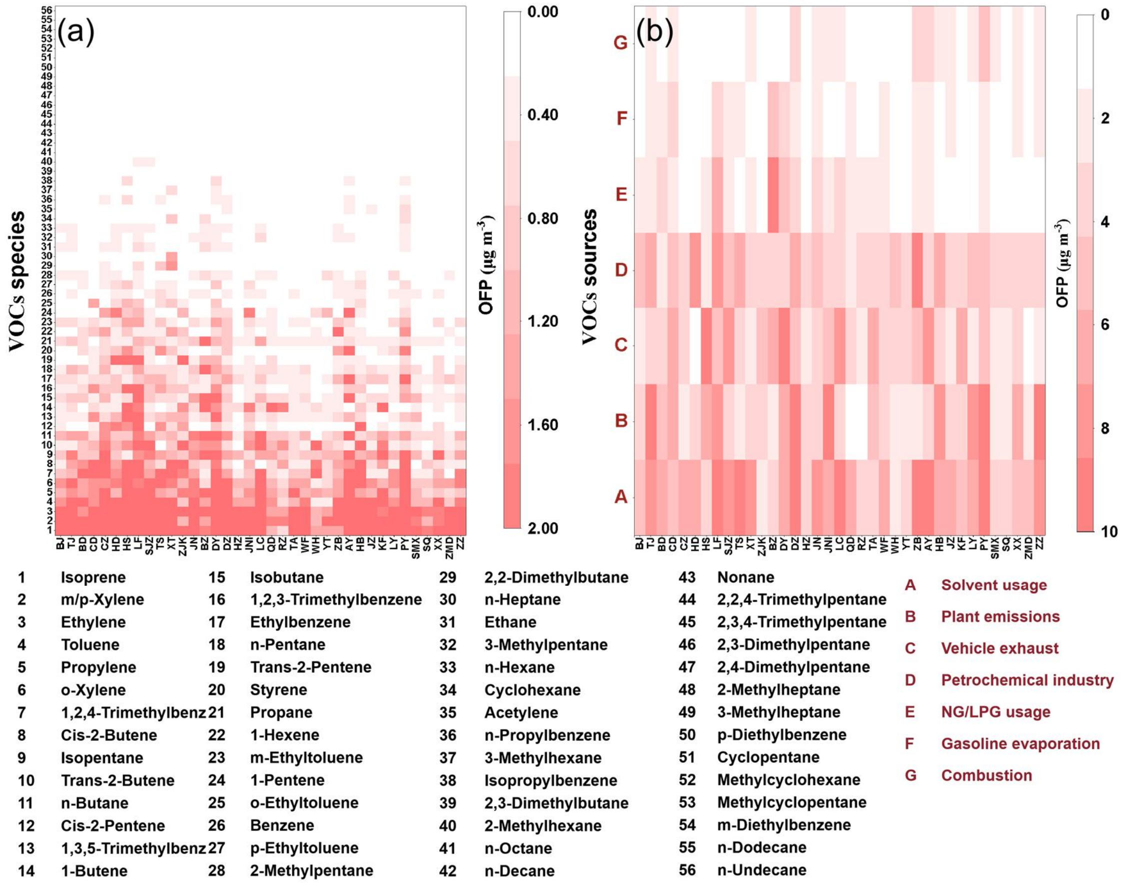

Fig. S8. Regional distributions of the calculated O_3 formation potential (OFP) of major reactive VOC species and their emission sources across 37 cities in the NCP region. (a) Averaged OFP for the 57 VOC species (*m*-xylene and *p*-xylene cannot be separated in the GC-MS and are treated as *m/p*-xylene); (b) The same as (a) but for the major VOC emission sources, which were identified using the Positive Matrix Factorization model. OFP is determined by multiplying the concentration of each VOC species by their respective maximum incremental reactivity (MIR) coefficients [46, 47]. In this study, the localized MIR values from each VOC species in Northern China were employed [47, 48].

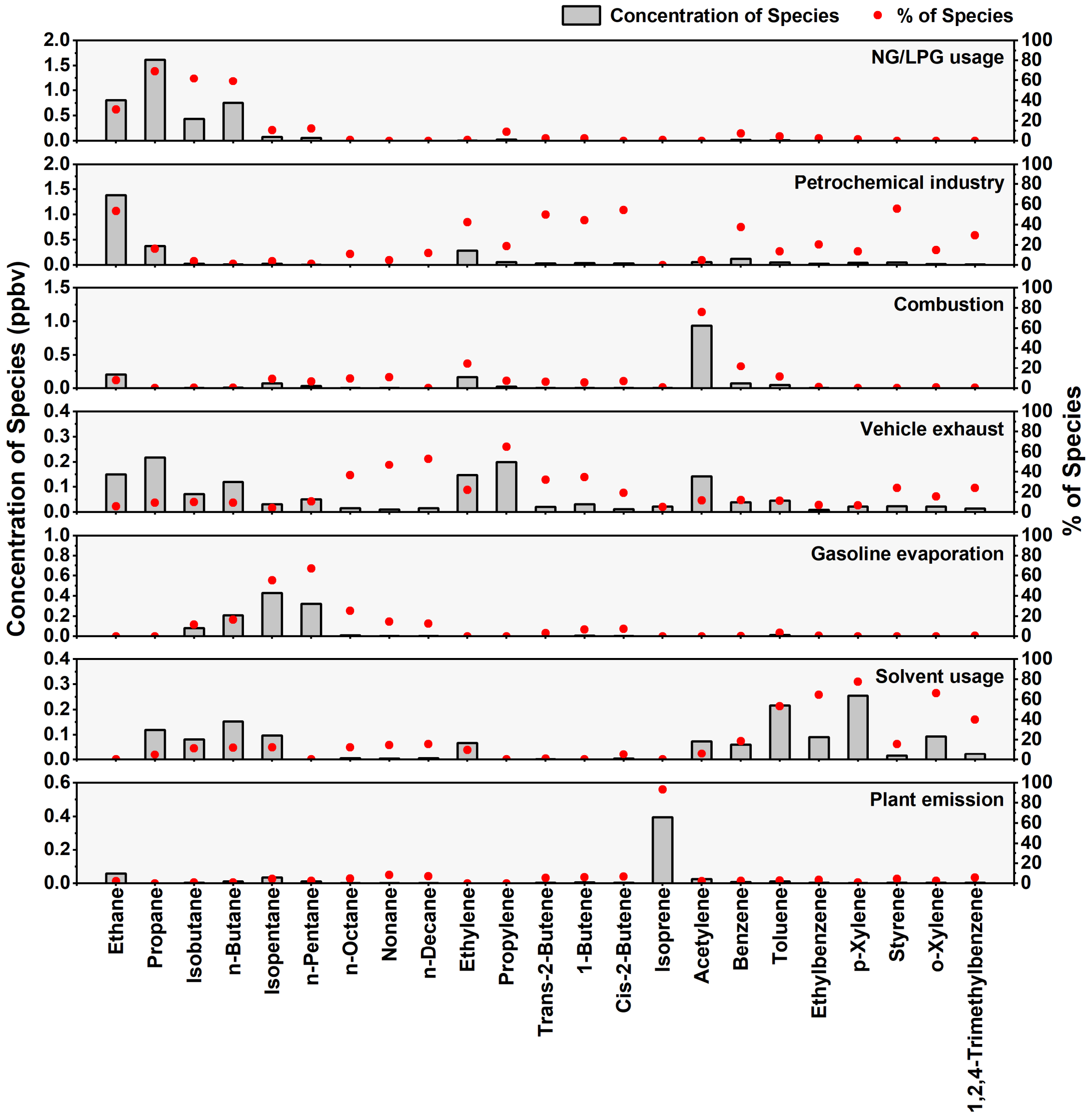

Fig. S9. VOC source profiles extracted from the PMF model for the 37 cities in the NCP region during June 1-August 31, 2021.

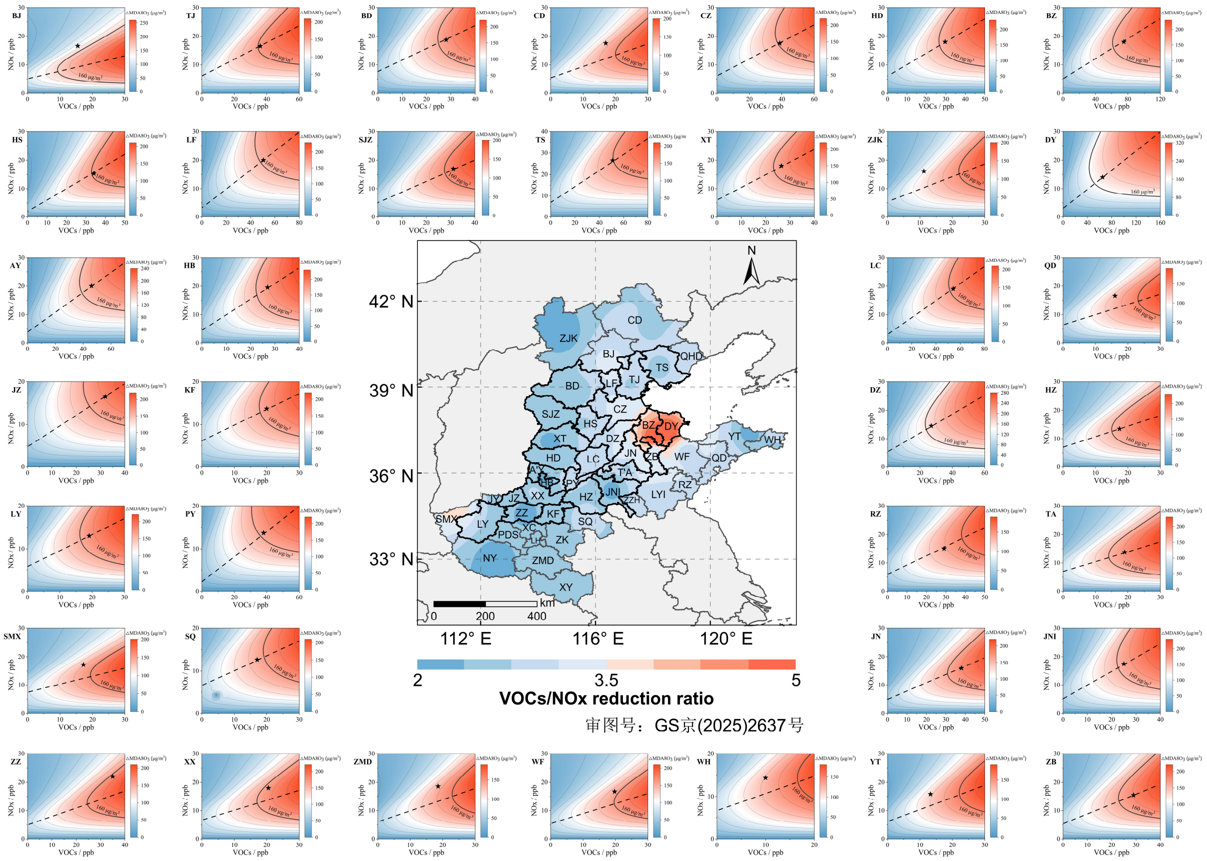

Fig. S10. Spatial distribution of the optimal AVOCs/NO_x reduction ratios for alleviating O_3 pollution, along with EKMA diagrams for 37 cities. In each EKMA diagram, the dashed ridge line represents the optimal AVOCs/NO_x reduction ratio. The black stars represent the annual evaluation concentrations of O_3 in 2021 in the EKMA diagrams.

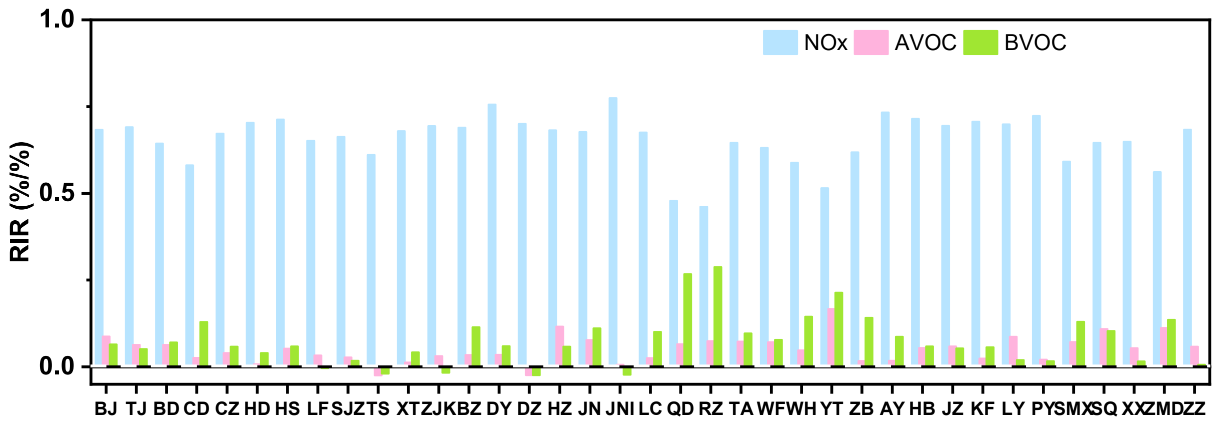

Fig. S11. RIR values of NO_x, AVOCs, and BVOCs in the "double-carbon" scenario for 2060. In each city, the RIR value of NO_x is more than twice that of VOCs, indicating that all cities fall under a NO_x-limited regime for O_3 formation.

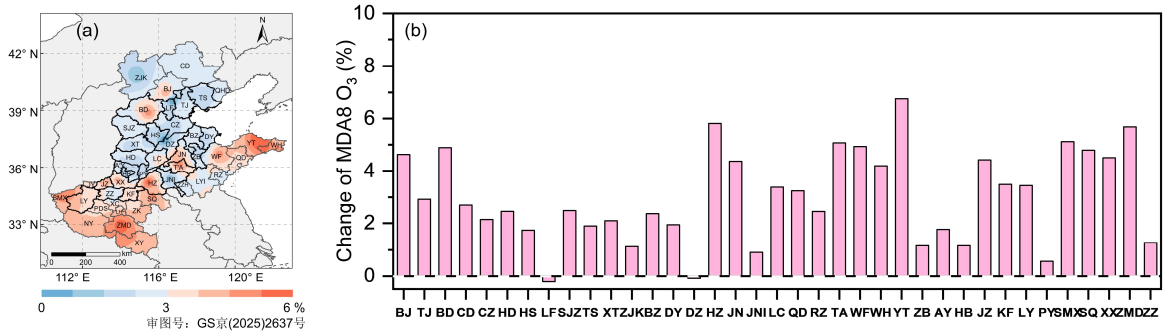

Fig. S12. Model-predicted percentage changes in MDA8 O_3 concentrations due to a 60% increase in BVOC emissions in the NCP region in 2060. (a) shows the spatial distribution of the percentage changes, and (b) presents the percentage changes for each city.

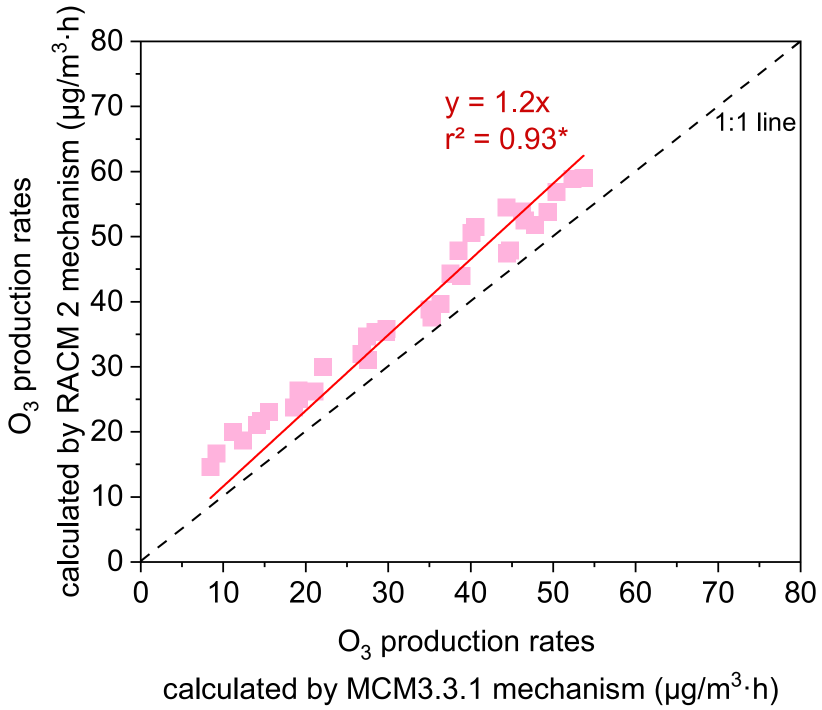

Fig. S13. Comparison of the model-simulated net O_3 production rates during daytime (07:00–19:00 local time) for the 37 cities in the NCP region. Each pink square represents the average value for an individual city. The models were built with the same configurations, with the only difference being the utilization of different atmospheric chemical mechanisms, i.e., the RACM2 and the MCM v3.3.1.

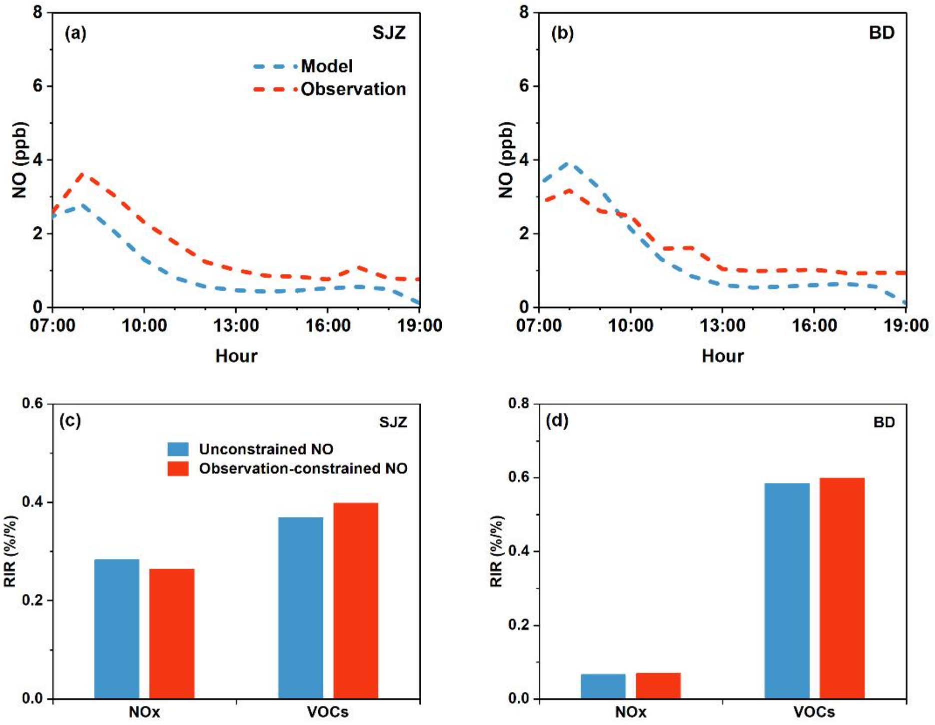

Fig. S14. Evaluation of OBM-simulated NO and impacts on RIR values. NO mixing ratios were directly observed in only two cities (i.e., SJZ and BD). (a–b) Comparison of modeled and observed diurnal NO profiles. (c–d) Comparison of calculated RIR values for simulations with and without NO constraints in the two cities.

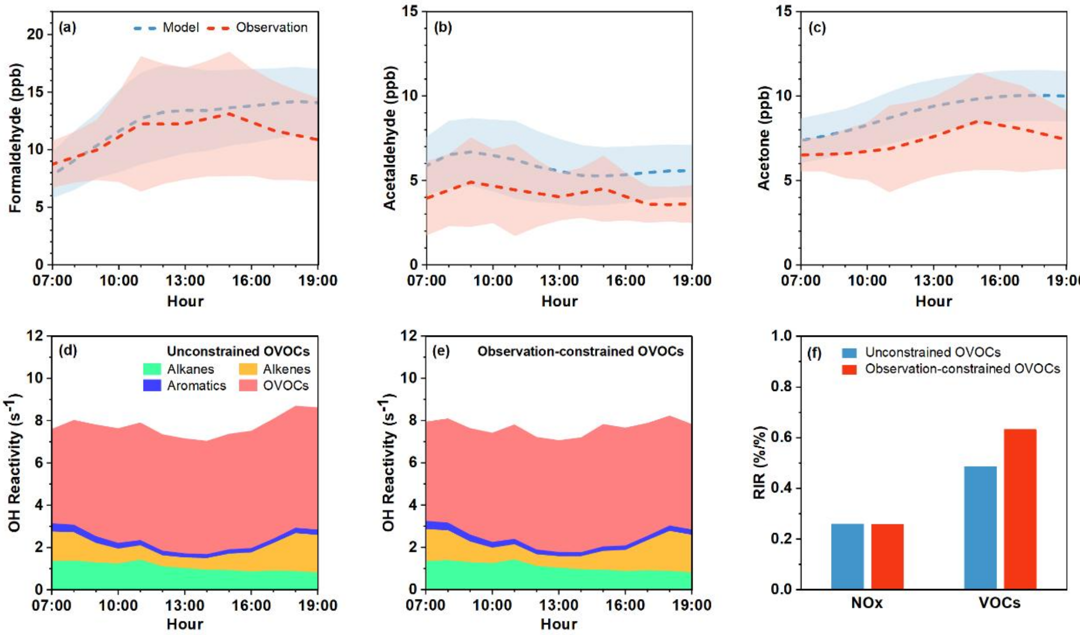

Fig. S15. Evaluation of the impacts of modeled OVOC concentrations on OH reactivity and RIR values. Data from the 2022 campaign in DY city (Fig. S6f), including 57 VOCs and 27 OVOC species. (a–c) Comparison of OBM-simulated OVOC concentrations with observations. Three major OVOCs were selected: formaldehyde, acetaldehyde, and acetone. Shaded areas represent the stand deviations. (d–e) OH reactivity contributions from major VOC groups (alkanes, alkenes, aromatics, and OVOCs) under unconstrained and observation-constrained OVOC conditions. (f) Comparison of the calculated RIR values for scenarios with and without OVOC constraints.

Table S1. Details of the 37 cities and concentration levels of major air pollutants in the NCP region from this study. For each city, the median values and the ranges (10th – 90th percentiles) of the pollutant levels are listed.

No.	Province/ Municipality	City	Abbreviation	MDA 8 O_3 (ppb)	NO_2 (ppb)	VOCs (ppb)
1	Beijing	Beijing	BJ	136 (78.2-178)	15.7 (10.8-21.4)	12.9 (7.67-19.5)
2	Tianjin	Tianjin	TJ	140 (97.1-189)	21.4 (14.0-32.2)	18.7 (12.5-28.7)
3	Hebei	Baoding	BD	150 (97.3-198)	19.4 (13.6-30.0)	13.4 (9.05-19.9)
4		Cangzhou	CZ	145 (101-185)	17.5 (11.5-27.1)	17.5 (11.7-30.7)
5		Chengde	CD	110 (74.2-142)	21.3 (17.6-24.7)	11. 5 (3.57-23.2)
6		Handan	HD	154 (90.4-215)	14.3 (8.08-23.8)	16.8 (11.4-24.3)
7		Hengshui	HS	147 (103-195)	16.7 (11.9-24.3)	14.1 (10.5-24.2)
8		Langfang	LF	146 (88.8-196)	21.5 (13.6-31.5)	25.1 (15.1-48.9)
9		Shijiazhuang	SJZ	154 (101-205)	19.7 (11.1-30.8)	18.4 (12.6-24.4)
10		Tangshan	TS	134 (95.2-194)	24.6 (15.7-35.6)	19.6 (6.42-30.6)
11		Xingtai	XT	156 (91.5-223)	19.4 (11.0-28.9)	17.7 (10.1-27.2)
12		Zhangjiakou	ZJK	127 (88-167)	13.7 (10.7-17.7)	9.96 (7.53-16.85)
13	Shandong	Binzhou	BZ	145 (101-208)	16.3 (10.0 -24.9)	31.9 (21.2-50.5)
14		Dezhou	DZ	154 (106-214)	13.3 (8.95-20.8)	22.3 (16.5-35.8)
15		Dongying	DY	135 (99.2-193)	12.3 (8.24-24.9)	41.8 (23.2-98.0)
16		Heze	HZ	139 (86.2-208)	14.0 (9.9-22.4)	7.63 (4.48-13.6)
17		Jinan	JN	162 (85.3-212)	20.3 (13.3-30.3)	16.1 (9.56-27.9)
18		Jining	JNI	145 (77.2-207)	14.5 (8.2-22.1)	12.0 (7.32-17.1)

19		Liaocheng	LC	147<br>(81.6-200)	19.2<br>(12.1-29.9)	19.4<br>(13.0-29.9)
20		Qingdao	QD	107<br>(69.2-172)	16.0<br>(8.83-27.7)	8.85<br>(2.89-19.0)
21		Rizhao	RZ	111<br>(68.1-179)	13.8<br>(6.22-30.8)	7.40<br>(2.46-13.9)
22		Taian	TA	164<br>(98.4-216)	17.1<br>(11.6-26.2)	10.6<br>(3.94-16.2)
23		Weifang	WF	124<br>(77.3-175)	14.2<br>(10.3-25.2)	9.98<br>(5.23-18.3)
24		Weihai	WH	111<br>(76.1-158)	11.9<br>(7.19-17.1)	5.75<br>(3.52-9.69)
25		Yantai	YT	120<br>(82.4-163)	16.4<br>(11.9-24.1)	6.73<br>(3.32-12.0)
26		Zibo	ZB	159<br>(88.5-211)	22.4<br>(14.2-29.7)	12.7<br>(7.85-20.0)
27		Anyang	AY	152<br>(89.3-216)	15.4<br>(8.85-29.9)	50.1<br>(28.4-68.6)
28		Hebi	HB	147<br>(81.0-217)	15.1<br>(9.32-28.7)	14.1<br>(10.0-18.4)
29		Jiaozuo	JZ	156<br>(82.2-221)	13.0<br>(7.37-19.6)	14.4<br>(8.85-20.0)
30		Kaifeng	KF	146<br>(80.1-203)	12.5<br>(8.00-21.5)	14.4<br>(9.87-17.8)
31		Luoyang	LY	146<br>(79.2-204)	15.0<br>(9.28-22.6)	12.2<br>(8.98-16.6)
32	Henan	Puyang	PY	141<br>(80-207)	13.8<br>(9.31-23.7)	22.8<br>(14.7-32.0)
33		Sanmenxia	SMX	140<br>(81.1-189)	16.0<br>(10.3-26.5)	10.8<br>(8.06-17.2)
34		Shangqiu	SQ	126<br>(75.1-207)	9.94<br>(5.62-18.1)	5.54<br>(3.378-9.91)
35		Xinxiang	XX	147<br>(83.3-217)	16.6<br>(10.6-31.9)	11.8<br>(7.28-18.9)
36		Zhengzhou	ZZ	153<br>(74-215)	17.8<br>(10.9-29.1)	13.0<br>(8.46-18.7)
37		Zhumadian	ZMD	114<br>(73.1-180)	10.8<br>(6.87-18.3)	6.41<br>(4.66-10.69)

Table S2 Median RIR_{NOx}/RIR_{VOCs} ratios for 37 cities in the NCP region.

Cities	RIR_{NOx}/RIR_{VOCs}	Cities	RIR_{NOx}/RIR_{VOCs}	Cities	RIR_{NOx}/RIR_{VOCs}
BJ	-0.016725	DY	0.9891159	AY	1.6523119
TJ	0.0504711	DZ	1.4290644	HB	0.8687543
BD	0.1144699	HZ	-0.110905	JZ	0.4100969
CZ	0.5330745	JN	-0.220217	KF	0.7139394
CD	-0.853668	JNI	1.1522252	LY	0.5511345
HD	0.8165228	LC	0.1914579	PY	1.5738718
HS	0.7707094	QD	-0.696936	SMX	-0.205277
LF	1.0940626	RZ	-0.790858	SQ	-0.082116
SJZ	0.7679345	TA	-0.228317	XX	0.2607734
TS	0.0691094	WF	-0.505871	ZZ	0.2980483
XT	0.5538678	WH	-0.226213	ZMD	0.0019805
ZJK	0.4349844	YT	-1.214048		
BZ	0.6476618	ZB	0.2268984		

Table S3. Summary of previous research on O_3–VOC–NO_x sensitivity for key cities in the NCP region. The literature review was conducted using "Web of Science" with the keywords "O_3 formation sensitivity" + "observation based model" + "China", and "O_3 formation sensitivity" + "air quality model" + "north china plain", with publications restricted to from 2010 to 2025. From the 88 relevant references identified, 16 papers reporting O_3 formation sensitivity at urban sites in the NCP region were selected.

Province/ Municipality	City	Period	O_3 formation sensitivity	Method
Beijing	Beijing [49]	August, 2005 to 2011	VOCs-limited	Observation-Based Model with SAPRC
	Beijing [50]	July 2014	VOCs-limited	Observation-Based Model with RACM2
	Beijing [28]	Summer, 2018	NO_x-VOCs co-limited	Observation-Based Model with MCMv3.2
	Beijing [27]	Summer and autumn, 2019	VOCs-limited	Observation-Based Model with MCMv3.3.1
	Beijing [26]	October, 2019	VOCs-limited during $PM_{2.5}$ pollution episodes; NO_x-VOCs co-limited during O_3 pollution episodes	Observation-Based Model with MCMv3.3.1
	Beijing [16]	1 May to 30 June, 2019	VOCs-limited	Observation-Based Model with MCMv3.3.1
	Beijing [16]	1 June to 31 August, 2020	VOCs-limited for most of the time, NO_x-VOCs co-limited during an O_3 pollution episode	Observation-Based Model with MCMv3.3.1
	Beijing [51]	April 2020 to March 2021	VOCs-limited during O_3 season; NO_x-VOCs co-limited O_3 pollution episodes	Observation-Based Model
Hebei	Baoding [25]	April to September, 2020	VOCs-limited	Observation-Based Model with MCMv3.3.1

	Langfang [25]	April to September, 2020	VOCs-limited	Observation-Based Model with MCMv3.3.1
	Tangshan [25]	April to September, 2020	VOCs-limited	Observation-Based Model with MCMv3.3.1
	Xingtai [25]	April to September, 2020	VOCs-limited	Observation-Based Model with MCMv3.3.1
Shandong	Jinan [52]	July to August, 2017	VOCs-limited on low O_3 days; NO_x-VOCs co-limited on O3 non-attainment days	Photochemical box model with MCM3.2
	Jinan [29]	2018-2019	VOCs-limited	Observation-Based Model with RACM2
	Dongying [31]	March to April, 2018	VOCs-limited	Framework for 0-D Atmospheric Modeling with MCM v3.3.1
	Zibo [53]	May to September, 2019	VOCs-limited for low-O_3 period; NO_x-VOCs co-limited for high-O_3 period	Framework for 0-D Atmospheric Modelling with MCMv3.3.1
	Tai'an [54]	May to June, 2019	NO_x-VOCs co-limited	Observation-Based Model with RACM2
Henan	Zhengzhou [17]	July, 2019	VOCs-limited on low O3 days; NO_x-VOCs co-limited on O_3 pollution episodes	Observation-Based Model with MCM, v3.3.1
	Zhengzhou [55]	June, 2019	VOCs-limited; NO_x-VOCs co-limited	Community Multiscale Air Quality (CMAQ) model v5.3.1
	Luoyang [56]	July to August, 2019	NO_x-VOCs co-limited	WRF-CHEM model (Version 3.5.1)
	Anyang [25]	April to September, 2020	VOCs-limited	Observation-Based Model with MCM, v3.3.1
	Hebi [25]	April to September, 2020	VOCs-limited	Observation-Based Model with MCM, v3.3.1

	Luoyang [25]	April to September, 2020	VOCs-limited	Observation-Based Model with MCM, v3.3.1
	Xinxiang [25]	April to September, 2020	VOCs-limited	Observation-Based Model with MCM, v3.3.1
	Jiaozuo [25]	April to September, 2020	VOCs-limited	Observation-Based Model with MCM, v3.3.1
	Jincheng [25]	April to September, 2020	VOCs-limited	Observation-Based Model with MCM, v3.3.1
Shanxi	Linfen [25]	April to September, 2020	VOCs-limited	Observation-Based Model with MCM, v3.3.1
	Yuncheng [25]	April to September, 2020	VOCs-limited	Observation-Based Model with MCM, v3.3.1
	Changzhi [30]	June, 2021	NO_x-VOCs co-limited for high-O_3 period	Framework for 0-D Atmospheric Modeling with MCM

Table S4. Summary of previous research on long-term O_3 formation sensitivity variations in the NCP region.

City/Region	Period	O_3 formation sensitivity	Method
Beijing [57]	2006–2020	Overall in the VOC-limited regime with a trend of moving towards the transition regime	MCM v3.3.1
NCP [58]	2005-2019	Areas with the VOCs-limited regime decrease from 26% to 2%; Areas with the VOC-NO_x co-limited regime increase from 60% to 71%; Areas with the NO_x-limited regime increase from 14% to 27%	Satellite data and air quality models
Jing-Jin-Ji [59]	2012-2016	VOC-limited regime reduced from 62.8% to 39.9%; VOC-NO_x co-limited regime increased from 25.0% to 46.2%	WRF-CMAQ model
NCP [60]	2016-2019	4.9% grids shifted from VOC-limited to VOC-NO_x co-limited regime, 9.6% grids shifted from VOC-NO_x co-limited to NO_x-limited regime	Satellite data and source-oriented chemical transport model
NCP [61]	2016-2019	Most NCP regions shift from the VOC-limited to the VOC-NO_x co-limited regime	Satellite data
Jing-Jin-Ji [62]	2013-2021	96% of the areas shifted to VOC-NO_x co-limited or NO_x-limited regimes in spring and summer	Satellite data

Table S5. Summary of previous research on optimal VOCs/NO_x reduction ratios for key cities in the NCP region. The references used in this table are also included in Table S3.

Province	City	Period	Optimal VOCs/NO_x reduction ratio	Method
Hebei	Baoding [25]	April to September, 2020	9:1	Observation-Based Model with MCMv3.3.1
	Langfang [25]	April to September, 2020	11:1	Observation-Based Model with MCMv3.3.1
	Tangshan [25]	April to September, 2020	5:1	Observation-Based Model with MCMv3.3.1
	Xingtai [25]	April to September, 2020	10:1	Observation-Based Model with MCMv3.3.1
Shandong	Zibo [53]	May to September, 2019	1:3	Framework for 0-D Atmospheric Modelling with MCMv3.3.1
	Jinan [29]	2018-2019	0.79:1	Observation-Based Model with RACM2
	Tai'an [54]	May to June, 2019	1:3	Observation-Based Model with RACM2
Henan	Anyang [25]	April to September, 2020	8:1	Observation-Based Model with MCM, v3.3.1
	Hebi [25]	April to September, 2020	8:1	Observation-Based Model with MCM, v3.3.1
	Luoyang [25]	April to September, 2020	8:1	Observation-Based Model with MCM, v3.3.1
	Luoyang [56]	July to August, 2019	3:1	WRF-CHEM model (Version 3.5.1)
	Xinxiang [25]	April to September, 2020	7:1	Observation-Based Model with MCM, v3.3.1
	Jiaozuo [25]	April to September, 2020	10:1	Observation-Based Model with MCM, v3.3.1

Shanxi	Jincheng [25]	April to September, 2020	11:1	Observation-Based Model with MCM, v3.3.1
	Linfen [25]	April to September, 2020	7:1	Observation-Based Model with MCM, v3.3.1
	Yuncheng [25]	April to September, 2020	9:1	Observation-Based Model with MCM, v3.3.1

Table S6 Multi-year trends in atmospheric NO_2 concentrations and NO_x emissions (2013–2020). Annual mean NO_2 concentrations are obtained from the China Ecological and Environmental Status Bulletin, and NO_x emissions are derived from the MEIC inventory. Values represent the averages across the 37 cities.

Year	NO_2 concentration (μg m^{-3})	NOx emission (tons/grid)
2013	47.68	32820.85
2014	45.83	30141.82
2015	43.97	27326.78
2016	41.78	26101.10
2017	42.22	24684.83
2018	38.60	24554.09
2019	36.46	24181.68
2020	32.58	22502.83
Decrease ratio	-31.68%	-31.44%

References:

1 Jiang Z, Wang S, Yan Y *et al*. Constructing the 3D spatial distribution of the HCHO/NO_2 ratio via satellite observation and machine learning model. *Environ Sci Technol* 2025; **59**: 4047–58.

2 Norris G, Duvall R, Brown S *et al*. EPA Positive Matrix Factorization (PMF) 5.0 Fundamentals and User Guide Prepared for the US. Environmental Protection Agency Office of Research and Development. *Washington, DC*. 2014; EPA/600/R-14/108.

3 Paatero P, Tapper U. Positive matrix factorization: A non-negative factor model with optimal utilization of error estimates of data values. *Environmetrics* 2006; **5**: 111–26.

4 Zhang H, Cheng S, Li H *et al*. Groundwater pollution source identification and apportionment using PMF and PCA-APCA-MLR receptor models in a typical mixed land-use area in Southwestern China. *Sci Total Environ* 2020; **741**: 140383.

5 Ryerson TB, Trainer M, Angevine WM *et al*. Effect of petrochemical industrial emissions of reactive alkenes and NOx on tropospheric ozone formation in Houston, Texas. *J Geophys Res Atmos* 2003; **108**: D08.

6 Watson JG, Chow JC, Fujita EM. Review of volatile organic compound source apportionment by chemical mass balance. *Atmos Environ* 2001; **35**: 1567–84.

7 Lv D, Lu S, Tan X *et al*. Source profiles, emission factors and associated contributions to secondary pollution of volatile organic compounds (VOCs) emitted from a local petroleum refinery in Shandong. *Environ Pollut* 2021; **274**: 116589.

8 Zheng H, Kong S, Yan Y *et al*. Compositions, sources and health risks of ambient volatile organic compounds (VOCs) at a petrochemical industrial park along the Yangtze River. *Sci Total Environ* 2020; **703**: 135505.

9 Liu Y, Shao M, Fu L *et al*. Source profiles of volatile organic compounds (VOCs) measured in China: Part I. *Atmos Environ* 2008; **42**: 6247–60.

10 Cai H, Xie SD. Tempo-spatial variation of emission inventories of speciated volatile organic compounds from on-road vehicles in China. *Atmos Chem Phys* 2009; **9**: 6983–7002.

11 Barletta B, Meinardi S, Rowland FS *et al*. Volatile organic compounds in 43 Chinese cities. *Atmos Environ* 2005; **39**: 5979–9.

12 Mo Z, Shao M, Lu S. Compilation of a source profile database for hydrocarbon and OVOC emissions in China. *Atmos Environ* 2016; **143**: 209–17.

13 Vestenius M, Hopke PK, Lehtipalo K *et al*. Assessing volatile organic compound sources in a boreal forest using positive matrix factorization (PMF). *Atmos Environ* 2021; **259**:

118503.

14 Goliff WS, Stockwell WR, Lawson CV. The regional atmospheric chemistry mechanism, version 2. *Atmos Environ* 2013; **68**: 174–85.

15 Jenkin ME, Young JC, Rickard AR. The MCM v3.3.1 degradation scheme for isoprene. *Atmos Chem Phys* 2015; **15**: 11433–59.

16 Li Y, Wu Z, Ji Y *et al.* Comparison of the ozone formation mechanisms and VOCs apportionment in different ozone pollution episodes in urban Beijing in 2019 and 2020: Insights for ozone pollution control strategies. *Sci Total Environ* 2024; **908**: 168332.

17 Wang X, Yin S, Zhang R *et al.* Assessment of summertime O_3 formation and the O_3-NO_x-VOC sensitivity in Zhengzhou, China using an observation-based model. *Sci Total Environ* 2022; **813**: 152449.

18 Xue LK, Wang T, Gao J *et al.* Ground-level ozone in four Chinese cities: precursors, regional transport and heterogeneous processes. *Atmos Chem Phys* 2014; **14**: 13175–88.

19 Jenkin ME, Saunders SM, Wagner V *et al.* Protocol for the development of the Master Chemical Mechanism, MCM v3 (Part B): tropospheric degradation of aromatic volatile organic compounds. *Atmos Chem Phys* 2003; **3**: 181–93.

20 Tan Z, Hofzumahaus A, Lu K *et al.* No evidence for a significant impact of heterogeneous chemistry on radical concentrations in the North China Plain in summer 2014. *Environ Sci Technol* 2020; **54**: 5973–9.

21 Li Y, Wang X, Wu Z, et al. Atmospheric nitrous acid (HONO) in an alternate process of haze pollution and ozone pollution in urban Beijing in summertime: Variations, sources and contribution to atmospheric photochemistry. *Atmos Res* 2021; **260**: 105689.

22 Xue L, Gu R, Wang T *et al.* Oxidative capacity and radical chemistry in the polluted atmosphere of Hong Kong and Pearl River Delta region: analysis of a severe photochemical smog episode. *Atmos Chem Phys* 2016; **16**: 9891–903.

23 Zhao M, Zhang Y, Pei C *et al.* Worsening ozone air pollution with reduced NO_x and VOCs in the Pearl River Delta region in autumn 2019: Implications for national control policy in China. *J Environ Manage* 2022; **324**: 116327.

24 Cardelino CA, Chameides WL. An observation-based model for analyzing ozone precursor relationships in the urban atmosphere. *J Air Waste Mana. Assoc* 1995; **45**: 161–80.

25 Shan D, Du Z, Zhang T *et al.* Variations, sources, and effects on ozone formation of VOCs during ozone episodes in 13 cities in China. *Front Env Sci* 2023; **10**: 1084592.

26 Jia C, Tong S, Zhang X *et al.* Atmospheric oxidizing capacity in autumn Beijing: Analysis of the O_3 and $PM_{2.5}$ episodes based on observation-based model. *J Environ Sci* 2023;

124: 557–69.

27 Han J, Liu Z, Hu B *et al*. Observations and explicit modeling of summer and autumn ozone formation in urban Beijing: Identification of key precursor species and sources. *Atmos Environ* 2023; **309**: 119932.

28 Liu X, Guo H, Zeng *et al*. Photochemical ozone pollution in five Chinese megacities in summer 2018. *Sci Total Environ* 2021; **801**: 149603.

29 Mu J, Zhang Y, Xia Z *et al*. Two-year online measurements of volatile organic compounds (VOCs) at four sites in a Chinese city: Significant impact of petrochemical industry. *Sci Total Environ* 2023; **858**: 159951.

30 Niu Y, Yan Y, Xing Y *et al*. Analyzing ozone formation sensitivity in a typical industrial city in China: Implications for effective source control in the chemical transition regime. *Sci Total Environ* 2024; **919**: 170559.

31 Lee Y, Huey L G, Wang Y *et al*. Photochemistry of Volatile Organic Compounds in the Yellow River Delta, China: Formation of O_3 and Peroxyacyl Nitrates. *J Geophys Res Atmos* 2021; **126**: e2021JD035296.

32 Zhao Y, Chen L, Li K *et al.* Atmospheric ozone chemistry and control strategies in Hangzhou, China: Application of a 0-D box model. *Atmos Res* 2020; **246**: 105109.

33 Liu T, Hong Y, Li M *et al*. Atmospheric oxidation capacity and ozone pollution mechanism in a coastal city of southeastern China: analysis of a typical photochemical episode by an observation-based model. *Atmos Chem Phys* 2022; **22**: 2173–90.

34 Chen G, Liu T, Chen J *et al*. Atmospheric oxidation capacity and O3 formation in a coastal city of southeast China: Results from simulation based on four-season observation. *J Environ Sci* 2024; **136:** 68–80.

35 Zhou J, Wang W, Wang Y *et al*. Intercomparison of measured and modelled photochemical ozone production rates: Suggestion of chemistry hypothesis regarding unmeasured VOCs. *Sci Total Environ* 2024; **951**: 175290.

36 Zhou J, Zhang C, Liu A *et al*. Measurement report: Vertical and temporal variability in the near-surface ozone production rate and sensitivity in an urban area in the Pearl River Delta region, China. *Atmos Chem Phys* 2024; **24**: 9805–26.

37 Ling ZH, Guo H, Lam SHM *et al*. Atmospheric photochemical reactivity and ozone production at two sites in Hong Kong: Application of a Master Chemical Mechanism–photochemical box model. *J Geophys Res Atmos* 2014; **119**: 10567–82.

38 Liu X, Lyu X, Wang Y *et al*. Intercomparison of O3 formation and radical chemistry in the past decade at a suburban site in Hong Kong. *Atmos Chem Phys* 2019; **19**: 5127–45.

39 Wang R, Wang L, Xue M *et al*. New insight into formation mechanism, source and control strategy of severe O3 pollution: The case from photochemical simulation in the Wuhan Metropolitan Area, Central China. *Atmos Res* 2023; **284**: 106605.

40 Lu X, Chen N, Wang Y *et al*. Radical budget and ozone chemistry during autumn in the atmosphere of an urban site in central China. *J Geophys Res Atmos* **122**: 3672–85.

41 Zhu J, Cheng H, Peng J *et al*. O_3 photochemistry on O_3 episode days and non-O_3 episode days in Wuhan, Central China. *Atmos Environ* 2020; **223**: 117236.

42 Zhan J, Zheng F, Xie R *et al*. The role of NO_x in Co-occurrence of O_3 and $PM_{2.5}$ pollution driven by wintertime east Asian monsoon in Hainan. *J Environ Manage* 2023; **345**: 118645.

43 Sebol AE, Canty TP, Wolfe GM *et al*. Exploring ozone production sensitivity to NOx and VOCs in the New York City airshed in the spring and summers of 2017–2019. *Atmos Environ* 2024; **324**: 120417.

44 Nussbaumer C M, Crowley J N, Schuladen J *et al.* Measurement report: Photochemical production and loss rates of formaldehyde and ozone across Europe. *Atmos Chem Phys* 2021; **21**: 18413–32.

45 Kanaya Y, Tanimoto H, Yokouchi Y *et al*. Diagnosis of Photochemical Ozone Production Rates and Limiting Factors in Continental Outflow Air Masses Reaching Fukue Island, Japan: Ozone-Control Implications. *Aerosol Air Qual Res* 2016; **16:** 430–41.

46 Carter W. P. L. Development of the SAPRC-07 chemical mechanism. *Atmos Environ* 2010; **44**: 5324–35.

47 Zhang Y, Xue L, Carter W P L *et al.* Development of ozone reactivity scales for volatile organic compounds in a Chinese megacity. *Atmos Chem Phys* 2021; **21**: 11053-68.

48 Zhang Y, Xue L, Mu J *et al.* Developing the Maximum Incremental Reactivity for Volatile Organic Compounds in Major Cities of Central-Eastern China. *J Geophys Res Atmos* 2022; **127**: e2022JD037296.

49 Zhang Q, Yuan B, Shao M *et al.* Variations of ground-level O_3 and its precursors in Beijing in summertime between 2005 and 2011. *Atmos Chem Phys* 2014; **14**: 6089-101.

50 Tan Z, Lu K, Jiang M *et al*. Daytime atmospheric oxidation capacity in four Chinese megacities during the photochemically polluted season: a case study based on box model simulation, *Atmos Chem Phys* 2019; **19**: 3493–513.

51 Liang S, Gao S, Wang S *et al*. Characteristics, sources of volatile organic compounds, and their contributions to secondary air pollution during different periods in Beijing, China. *Sci Total Environ* 2023; **858**: 159831.

52 Lyu X, Wang N, Guo H *et al*. Causes of a continuous summertime O_3 pollution event in Jinan, a central city in the North China Plain. *Atmos Chem Phys* 2019; **19**: 3025–42.

53 Li L, Zheng Z, Xu B *et al*. Investigation of O_3-precursor relationship nearby oil fields of Shandong, China. *Atmos Environ* 2023; **294**: 119471.

54 Li Y, Ye C, Ma X *et al*. Radical chemistry and VOCs-NOx-O_3-nitrate sensitivity in the polluted atmosphere of a suburban site in the North China Plain. *Sci Total Environ* 2024; **947**: 174405.

55 Su F, Xu Q, Yin S *et al*. Contributions of local emissions and regional background to summertime ozone in central China. *J Environ Manage* 2023; **338**: 11777.

56 Sun J, Shen Z, Wang R *et al*. A comprehensive study on ozone pollution in a megacity in North China Plain during summertime: Observations, source attributions and ozone sensitivity. *Environ. Int.* 2021; **146**: 106279.

57 Wang W, Li X, Cheng Y *et al*. Ozone pollution mitigation strategy informed by long-term trends of atmospheric oxidation capacity. *Nat Geosci* 2024; **17**: 20-25.

58 Li R, Xu M, Li M *et al*. Identifying the spatiotemporal variations in ozone formation regimes across China from 2005 to 2019 based on polynomial simulation and causality analysis. *Atmos Chem Phys* 2021; **21**: 15631–46.

59 Wang N, Lyu X, Deng X et al. Aggravating O_3 pollution due to NOx emission control in eastern China. *Sci Total Environ* 2019; **677**: 732–44.

60 Zhu S, Ma J, Wang S *et al*. Shifts of formation regimes and increases of atmospheric oxidation led to ozone increase in North China Plain and Yangtze River Delta from 2016 to 2019. *J Geophys Res Atmos* 2023; **128**: e2022JD038373.

61 Wang W, van der A R, Ding J *et al*. Spatial and temporal changes of the ozone sensitivity in China based on satellite and ground-based observations. *Atmos Chem Phys* 2021; **21**: 7253–69.

62 Wang Y, Zhao Y, Liu Y *et al.* Sustained emission reductions have restrained the ozone pollution over China. *Nat Geosci* 2023; **16**: 967-974.
